# Galactosylated liposomes for targeted encapsulation and enhanced cytotoxicity of Mistletoe Lectin, an antitumoral type 2 ribosome-inactivating protein

**DOI:** 10.1016/j.ijpx.2025.100392

**Published:** 2025-09-12

**Authors:** Josanna Kaufmann, Eray Cetin, Tiana Kraus, Harden Rieger, Gero Leneweit

**Affiliations:** a ABNOBA GmbH, 75223 Niefern-Öschelbronn, Germany; b Carl Gustav Carus-Institute, 75223 Niefern-Öschelbronn, Germany; c Department of Chemical and Process Engineering, Institute of Mechanical Process Engineering and Mechanics, Karlsruhe Institute of Technology, 76131 Karlsruhe, Germany

**Keywords:** Drug delivery systems, Liposomes, Cancer therapy, Lectin, Protein encapsulation

## Abstract

The development of efficient liposomal encapsulations of proteins for pharmaceutical applications is limited by several factors: their high molecular weight, interactions with surrounding substances, or the generally lower stability compared to small molecules. In this work, various liposomal formulations were prepared using the thin-film hydration method followed by extrusion, to investigate their suitability for the encapsulation of the plant-derived antitumoral mistletoe lectin-1 (ML-1). This can be significantly optimized by exploiting its preferential binding to galactose-containing structures, such as modified lipids integrated into the liposomal bilayer. Incorporation of the galactosylated lipid DSPE-PEG2k-Gal into the membrane significantly enhanced the overall recovery rate and encapsulation efficiency of ML-1, attributed to its affinity for the functionalized component. Compared to non-functionalized liposomes, a 2-fold to 4-fold increase in percentage encapsulation efficiency was observed. The galactosylated lipid optimized the ratio of encapsulated to surface-adsorbed ML-1 and facilitated its preferential localization within the core of the liposomes. A strong correlation was identified between the number of entrapped ML-1 molecules per liposome and the degree of galactosylation. The formulations demonstrated high in vitro cytotoxicity, as exemplified with murine colon-26 carcinoma cells, with the galactose-functionalized liposomes achieving an IC_50_ value comparable to free ML-1. This strategy presents significant potential for developing more efficient and targeted liposomal formulations of pharmaceutical proteins with specific affinities to tailored lipid components, advancing drug delivery technologies, and improving therapeutic options for cancer treatment.

## Highlights

• Optimized liposomal formulation achieved for a high molar weight antitumoral lectin.
• Galactosylated lipids are proven to enable affinity-mediated lectin encapsulation.
• Galactosylation increases encapsulation efficiency 2-fold to 4-fold.
• Lectin used as drug can be localized within the liposomal core.
• Cytotoxic activity of affinity-bound lectin is equal to dissolved lectin.

## Graphical abstract

**Figure:**
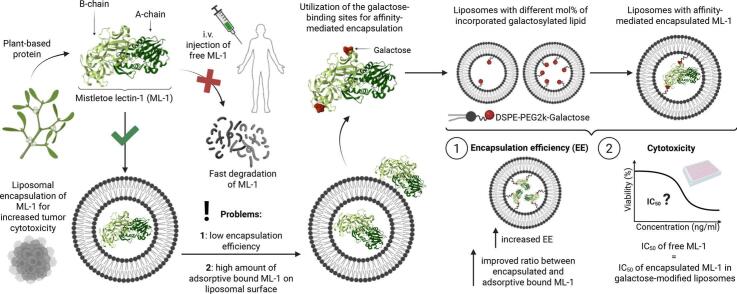

## 1 Introduction

Colorectal carcinomas (CRC) are the third most prevalent cancer worldwide, representing approximately 10 % of all cancer cases (Sung et al., 2021). These tumors exhibit a high propensity for developing resistance to conventional cytostatic therapies, significantly compromising treatment efficacy (Ashique et al., 2024). Multi-drug resistance (MDR) to chemotherapeutic agents is mediated by mechanisms such as the upregulation of efflux transporters, including P-glycoproteins, which reduce intracellular drug concentrations, and the overexpression of degrading enzymes like dihydropyrimidine dehydrogenase, which catalyze the inactivation of chemotherapeutics (Begicevic and Falasca, 2017; Dean et al., 2005; Salonga et al., 2000). Additionally, tumor cells may upregulate DNA repair mechanisms, such as nucleotide excision repair, to counteract the induced DNA damage (Arnould et al., 2003). The overexpression of apoptosis inhibitors, including Bcl-2, Bcl-XL, and c-FLIP, can also contribute to resistance to both chemo- and radiotherapy (Hayward et al., 2004; Longley et al., 2006; Marin et al., 2012).

Various strategies are currently explored to improve CRC therapy, with nanomedicine standing out as a particularly promising approach (Jain and Bhattacharya, 2023; Kumar et al., 2023). Advanced drug delivery systems are being developed to enhance the targeted delivery of therapeutics while minimizing systemic side effects. Nanocarriers provide a significant advantage by protecting drugs from premature degradation and ensuring precise delivery to the intended site of action (Fatima et al., 2018; Fenske et al., 2008). Liposomes, defined as spherical vesicles composed of one or more phospholipid bilayers, represent a prominent class of nanocarriers. Due to their amphiphilic properties, they can carry hydrophilic drugs in their aqueous center and hydrophobic drugs within the bilayer. Liposomal formulations can decrease systemic drug toxicity by optimizing pharmacokinetics and improving intracellular drug concentration compared to free drugs (Sercombe et al., 2015). The encapsulation of chemotherapeutic agents within liposomal carriers can attenuate their distribution to non-targeted healthy tissues, thereby enhancing the therapeutic index (Allen et al., 2005). Liposomes exploit the enhanced permeability and retention (EPR) effect to preferentially accumulate in tumor tissues (Maeda et al., 2000). For further optimization, liposomes can be surface-modified to enable a more efficient uptake due to the cell-specific surface ligands that target overexpressed structures on tumor cells (Lehner et al., 2013; Rivankar, 2014). Encapsulation of chemotherapeutic agents such as doxorubicin (DOX) in functionalized liposomes targeting MDR colon cells resulted in increased drug accumulation, apoptosis, autophagy induction, and downregulation of apoptosis inhibitors compared to the free drug (Kang et al., 2017). Liposomal chemotherapeutics significantly improve the efficiency of drug delivery and increase the quality of life. However, they do not solve the persistent problem of resistance development in tumors and accordingly, they fail to prolong overall survival or progression-free survival (Petersen et al., 2016; Zhang et al., 2021). This limitation underscores the importance of exploring alternative therapeutic strategies and agents with different mechanisms of action to counteract resistance and to improve tumor targeting using liposomal ligands.

Mistletoe lectin-1 (ML-1) is a protein of significant interest for therapeutic applications, particularly in the development of cancer treatments, due to its potent cytotoxicity and immunostimulatory effects (Majeed et al., 2021). It is a heterodimeric glycoprotein derived from *Viscum album* with a molecular weight of 63 kDa (Krauspenhaar et al., 1999). Classified as a type II ribosome-inactivating protein (type II RIP), it belongs to the enzyme class of polynucleotide adenosine glycosidases found in plants (Barbieri et al., 1997). The toxicity is mediated by the interaction of the carbohydrate-binding domain of the mistletoe lectin B-chain (MLB) and the catalytic domain of the mistletoe lectin A-chain (MLA). In vivo studies have already demonstrated that lectin-modified surfaces can enhance the liposomal uptake in the intestinal tissue (Makhlof et al., 2021). MLB functions as a cellular recognition unit with two binding sites, α1 and γ2, that specifically interact with the hydroxyl groups of D-galactose or galactose-containing structures, such as di- and oligosaccharides (Mikeska et al., 2005; Wu et al., 1992). The expression level of galactose on the cell surface is closely associated with the progression of CRC, with significant upregulation observed in its tumor stage II, highlighting its potential as a therapeutic target for ML-1 (Zhao et al., 2018). Further investigations into the binding capacity of ML-1 have demonstrated its higher affinity for gangliosides and glycoproteins on the cell surface, primarily targeting structures with terminal Neu5Acα2–6Galβ1-4GlcNAcβ residues (Mohammed and Ferry, 2021; Müthing et al., 2002; Müthing et al., 2004). Müthing et al. first identified three alpha-2,6-sialyllactosamine (CD75s) receptors — IV6nLc4Cer, VI6nLc6Cer, and VIII6nLc8Cer - as the cellular ligands of ML-1 (Müthing et al., 2005). The expression of the CD75s motifs is upregulated in CRC and is associated with tumor progression and advanced stages of the disease (Costa-Nogueira et al., 2009; Elpek et al., 2002; Villar-Portela et al., 2011). Shilova et al. extended the specification as the two binding sites of MLB differ in their ligand recognition mechanisms, also one showing a higher affinity for 6-O-sulfated structures, such as O-sulfated lactose, 6-O-sulfated *N*-acetyllactosamine and 6-O-sulfated LacdiNAc (Shilova et al., 2022). Upon MLB binding to glycosylated cell structures, the toxin is internalized via endocytosis and transported through the Golgi network to the endoplasmic reticulum (Beztsinna et al., 2018; Citores and Ferreras, 2023). The catalytic MLA inactivates protein synthesis by deadenylating the adenosine residue of the 28S rRNA within the eukaryotic 60S ribosomal subunit (Endo et al., 1988). Additionally, the MLA is involved in several apoptotic pathways, including downregulating of the anti-apoptotic protein Bcl-2, inhibiting telomerase activity, activating various caspase residues to promote intrinsic apoptosis, and disrupting mitochondrial membrane potential (Coulibaly and Youan, 2017; Kim et al., 2000; Yau et al., 2015). ML-1 already has shown high cytotoxic potential against the in vitro colon carcinoma cell line CT26 with a IC_50_ value of 36.6 ng/ml (Beztsinna et al., 2018). Thus, ML-1 serves a dual role: acting as a ligand for various overexpressed cell surface structures to facilitate internalization and strongly inducing subsequent antitumoral effects. This identifies ML-1 as a promising protein-based candidate for CRC therapy, with its functionality primarily relying on the disruption of biosynthesis rather than inhibition of DNA replication seen with chemotherapeutics. The primary focus for the therapeutic application of ML-1 is the development of an optimized drug delivery system to enhance its circulation time after intravenous injection and effectively target tumor tissue.

The objective of this study was to integrate functionalized lipids into the liposomal phospholipid bilayer to selectively enhance the encapsulation efficiency of ML-1 via its affinity to galactosylated membrane components at the inner leaflet of the liposomal bilayers. Affinity binding of ML-1 to the liposomal surface was studied in parallel with respect to overexpressed tumor cell surface structures. This liposomal formulation was tested regarding the release of ML-1 from the affinity to galactosylated components at the inside and outside of the liposomal membrane. For this aim, the drug's high cytotoxic activity was tested, thereby optimizing its therapeutic potential for effective CRC treatment.

## 2 Material and methods

### 2.1 Material

The phospholipids 1,2-Dimyristoyl-*sn*-glycero-3-phosphocholine (DMPC) and 2-Distearoyl-*sn*-glycero-3-phosphoethanolamine-N-(polyethylene glycol)2000 (PEG2k) were supplied by LIPOID GmbH (Ludwigshafen, Germany) and 1,2-Distearoyl-*sn*-glycero-3-phosphoethanolamine-N-(polyethylene glycol)2000-galactose (PEG2k-Gal) by Biopharma PEG Scientific Inc. (Watertown, NY, USA). Mistletoe lectin-1 (ML-1) was obtained from ABNOBA GmbH (Niefern-Öschelbronn, Deutschland) and cholesterol, ethanol, phosphate buffered saline (PBS) and Triton® X 100 were purchased from Carl Roth GmbH & Co. KG (Karlsruhe, Germany).

### 2.2 Preparation of liposomes

The different liposomal formulations were prepared using the thin-film hydration method followed by extrusion. Stock solutions of the lipids DMPC, cholesterol, PEG2k and PEG2k-Gal were prepared in ethanol and mixed in a round-bottom flask in ratios corresponding to the desired liposomal compositions (Table 1). The study encompassed the preparation of non-galactosylated liposomes (Control and PEG2k) and galactosylated liposomes with defined molar ratios of PEG2k-Gal (Gal2.5, Gal5, and Gal10), analyzed with and without encapsulated ML-1. The ethanol of each lipid mixture was evaporated using a rotary evaporator, which led to the formation of a thin lipid film. The dried lipids were resuspended in either 5 ml of 10 mM PBS pH 7.4 or in a ML-1 solution with $c_{\mathrm{ML}-1}$*=* 0.25 mg/ml in PBS, achieving a final lipid concentration of $c_{\mathrm{Lipid}}\;$= 5 mM. The subsequent extrusion process was carried out using a Lipex® extruder (Northern Lipids Inc., Burnaby, BC, Canada). The liposome dispersion was extruded through 100 nm track-etched polycarbonate membranes (Whatman™, Cytiva, Marlborough, MA, United States) for 10 passes to ensure uniform vesicle size and distribution. Non-encapsulated protein was purified by dialysis using a Spectra-Por® Float-A-Lyzer® G2 (Repligen Corp., Waltham, MA, United States) with a 300 kDa molecular weight cutoff. Dialysis of the drug containing samples were conducted against a 300-fold excess of PBS, whereas the buffer was replaced after 2 h and 4 h. After 24 h of dialysis, all liposomal samples were subjected to sterile filtration through a 0.22 μm membrane.

**Table 1:** Compositions of the liposomal samples.

	Control / Control + ML-1	PEG2k / PEG2k + ML-1	Gal2.5 / Gal2.5 + ML-1	Gal5 / Gal5 + ML-1	Gal10 / Gal10 + ML-1
Composition	Molar ratio (mol%)				
DMPC	60	55	52.5	50	50
Cholesterol	40	40	40	40	35
PEG2k	–	5	5	5	5
PEG2k-Gal	–	–	2.5	5	10

### 2.3 Characterization of liposomes

Characterization of the liposomal size (*Z*-Average), the polydispersity index (PDI) and the zeta potential (ZP) was done via dynamic light scattering with the ZetaSizer ZS90 (Malvern Instruments, Worcestershire, United Kingdom). To evaluate the development of the size and homogeneity during the extrusion, the liposomes were measured after each membrane passage. For the 4-weeks storage stabilities, liposomal formulations were stored under sterile conditions at 4 °C and the Z-Average and PDI were measured weekly.

### 2.4 Quantification of liposomes

Following Stewart et al. the phospholipid concentration was determined colorimetrically at 488 nm (Stewart, 1980). For liposomal samples containing DMPC along with PEG2k and optionally PEG2k-Gal, the calibration curve was generated using a lipid mixture with the same molar ratios as those present in the liposomal formulation. This adjustment was carefully implemented to compensate for potential signal deviations and to enable more accurate and representative quantification of phospholipids. Calibration curves specific to each liposomal formulation are provided in the Supplementary Material (Fig. S5a-e). A strong linear correlation was observed between changes in the slope of the calibration curves and the varying proportions of DMPC in the liposomal compositions (Fig. S5f; Pearson's r_p_ = 0.979, *p* = 0.02). Due to the hydrophilic character of PEG2k and the galactosylation of PEG2k-Gal, their solubility in chloroform is expected to be reduced. In addition, the bulky and flexible nature of PEG chains introduces steric hindrance that can impair or even prevent complex formation between the phosphate group of PEGylated lipids and ammonium molybdate. As a result, phosphomolybdate complex formation is inefficient or negligible for PEGylated components. Only the non-PEGylated DMPC component readily partitions into the organic phase and contributes significantly to the absorbance signal. This selective extraction leads to a decreased apparent signal as the molar fraction of DMPC decreases and the content of PEGylated components increases. Therefore, in liposomal systems comprising DMPC, cholesterol, PEG2k, and PEG2k-Gal, the signal detected by the Stewart assay predominantly reflects the DMPC content. Based on the initially applied DMPC concentration, the lipid recovery rate (${\mathrm{RR}}_{\mathrm{Lipid}})\;$after the manufacturing process was calculated for each formulation.

The quantification of PEG2k-Gal was performed using the Amplex Red® Galactose/Galactose Oxidase Kit (Thermo Fisher Scientific Inc., Waltham, MA, USA), with modified standard solution. Galactose standards were replaced by the galactosylated lipid PEG2k-Gal to generate an adjusted calibration curve. To assess the distribution of PEG2k-Gal within the liposomal bilayer, measurements were first taken of the intact liposomes to determine the outer concentration. To evaluate the galactose content within the inner part of the liposomes, the samples were first disrupted with 5 % (*v*/v) Triton® X 100 in the assay buffer, allowing for the measurement of total galactose content. The calibration curve for the degraded liposomal sample was modified by incorporating an identical quantity of Triton® X 100 into the PEG2k-Gal assay standards. The inner concentration of PEG2k-Gal was subsequently derived by subtracting the outer from the total concentration. According to the quantified total, inner and outer concentration, the percentage distribution per outer and inner lipid layer ($P_{\mathrm{out}}$ $/P_{\mathrm{in}}$) was determined.

### 2.5 Quantification of ML-1

Quantification of ML-1 in the final samples was performed using the Pierce™ BCA Protein Assay Kit (Thermo Fisher Scientific Inc., Waltham, MA, USA). Blank (drug-free) liposomes with identical phospholipid concentration and composition of the drug-containing samples were used as a correction factor for absorbance measurements. Liposomes were analyzed both with and without the addition of 5 % (v/v) Triton® X 100 in the reaction buffer to differentiate between the surface-adsorbed ML-1 concentration $\left({c_{\mathrm{ML}-1}}_{\mathrm{SA}}\right)$ and the encapsulated ML-1 concentration (${c_{\mathrm{ML}-1}}_{E})$. Triton® X 100 induces liposome disruption, facilitating the release of encapsulated ML-1 and enabling the measurement of the total recovered protein concentration $\left({c_{\mathrm{ML}-1}}_{R}\right)$ in the final sample. The ${c_{\mathrm{ML}-1}}_{E}$ was determined by subtracting the ${c_{\mathrm{ML}-1}}_{\mathrm{SA}}$ from the ${c_{\mathrm{ML}-1}}_{R}$. The percentage of drug recovered is termed the recovery rate $({\mathrm{RR}}_{\mathrm{ML}-1}$) and was calculated relative to the initial concentration of ML-1 $\left(c_{\mathrm{ML}-1_{I}}\right)$ used for resuspending the lipid film. The encapsulation efficiency (EE) was calculated as the EE of ${c_{\mathrm{ML}-1}}_{E}$ relative to the $c_{\mathrm{ML}-1_{R}}$, which is further expressed as ${\mathrm{EE}}_{R}$, according to Eq. (1a), and the ${c_{\mathrm{ML}-1}}_{E}$ related to the $c_{\mathrm{ML}-1_{I}}$ as ${\mathrm{EE}}_{I}$, see Eq. (1b). Additionally, to the determination of the EE, the amount of surface-adsorbed ML-1 (SA) was expressed as the relative percentage of ${c_{\mathrm{ML}-1}}_{\mathrm{SA}}$ to $c_{\mathrm{ML}-1_{R}}$, represented by ${\mathrm{SA}}_{R}$, according to Eq. (2a), and relative to $c_{\mathrm{ML}-1_{I}}$ presented as ${\mathrm{SA}}_{I}$, using Eq. (2b). To compare the liposomal loading of ML-1 per formulation, the ratiobetween ${\mathrm{SA}}_{R}\;$to ${\mathrm{EE}}_{R}$ was calculated, which is defined as $R_{{\mathrm{SA}}_{R}/{\mathrm{EE}}_{R}}$. For the specification of the improvement in ML-1 encapsulation using the galactosylated lipids compared to non-galactosylated formulations the Increase factor $\left(\mathrm{IF}\right)$ was estimated using Eq. (3), by including the ${\mathrm{EE}}_{R}$ of the galactose-containing (${\mathrm{EE}}_{\mathrm{Gal}}$) and the galactose-free liposomes (${\mathrm{EE}}_{\mathrm{Gal}-\mathrm{free}}$) into the calculation.

(1a) $${\mathrm{EE}}_{R}=\frac{{c_{\mathrm{ML}-1}}_{E}}{c_{\mathrm{ML}-1_{R}}}*100$$

(1b) $${\mathrm{EE}}_{I}=\frac{{c_{\mathrm{ML}-1}}_{E}}{c_{\mathrm{ML}-1_{I}}}*100$$

(2a) $${\mathrm{SA}}_{R}=\frac{{c_{\mathrm{ML}-1}}_{\mathrm{SA}}}{c_{\mathrm{ML}-1_{R}}}*100$$

(2b) $${\mathrm{SA}}_{I}=\frac{{c_{\mathrm{ML}-1}}_{\mathrm{SA}}}{c_{\mathrm{ML}-1_{I}}}*100$$

(3) $$\mathrm{IF}=\frac{{\mathrm{EE}}_{\mathrm{Gal}}}{{\mathrm{EE}}_{\mathrm{Gal}-\mathrm{Free}}}$$

To evaluate the liposomal formulation regarding to the number of encapsulated ML-1 proteins per liposome, the average number of lipids $\left(N_{\mathrm{Lipids}}\right)$ per liposome was initially calculated with Eq. (5) according to mathematical approach demonstrated by Mozafari et al. (2021) with a bilayer thickness (h) of 5 nm (Hofsäss et al., 2003, Leonenko et al., 2004). Due to the lipid mixture, the model was modified using average headgroup area ($a_{\mathrm{avg}})$ presented in Eq. (4) (Hung et al., 2007; Mackay et al., 2019), based on the molar ratios (x) of the individual lipid components $\left(i\right)$, using reported headgroup areas $\left(a\right)$ of 61 Å^2^ for DMPC (Kucerka et al., 2005), 30 Å^2^ for cholesterol (Falck et al., 2004) and 140 Å^2^ for the lipid DSPE-PEG2k (Stepniewski et al., 2011). In the absence of specific experimental data for the headgroup area of DSPE-PEG2k-Gal, we have assumed the value reported for DSPE-PEG2k (140 Å^2^), to be applicable to the galactosylated variant as well. This assumption is justified by the close structural similarity between the two molecules, differing only in the terminal group.

(4) $$a_{\mathrm{avg}}=\sum_{i}x_{i}*a_{i}$$

(5) $$N_{\mathrm{Lipids}}=\frac{4\pi }{a_{\mathrm{avg}}}*\left[{\left(\frac{d}{2}\right)}^{2}+{\left(\frac{d}{2}-h\right)}^{2}\right]$$

This enabled the determination of the total number of liposomes per ml $\left(N_{\mathrm{Liposomes}}\right)$ based on the lipid concentration in the suspension using Eq. (6) and Avogadro's number *N*_*A*_. Additionally, the total encapsulated aqueous interior volume per ml liposomal suspension $\left(V_{\mathrm{captured}}\right)\;$was calculated in relation to the number of liposomes per ml according to Eq. (7). By calculating the number of ML-1 proteins per ml $\left(N_{\mathrm{ML}-1}\right)$ using the ${c_{\mathrm{ML}-1}}_{E}$ or ${c_{\mathrm{ML}-1}}_{\mathrm{SA}}$ the number of encapsulated $(N_{\mathrm{In}}$) and surface-adsorbed $(N_{\mathrm{out}}$) ML-1 molecules per one liposome was calculated by Eq. (8), resulting in the total number of ML-1 molecules per one liposome $(N_{\mathrm{Total}}$).

(6) $$N_{\mathrm{Liposomes}}=\frac{c_{\mathrm{Lipid}}∙N_{A}}{N_{\mathrm{Lipids}}\;}$$

(7) $$V_{\mathrm{captured}}=N_{\mathrm{Liposomes}}∙V_{\mathrm{encapuslated}}$$

(8) $$N_{\mathrm{Total}}=\frac{N_{\mathrm{ML}-1}}{N_{\mathrm{Liposomes}}}$$

### 2.6 In vitro analysis

#### 2.6.1 Cell culture and reagents

The murine colon carcinoma cell line colon-26 was purchased from CLS Cell Line Service GmbH (Eppelheim, Germany). Cells were cultured at 37 °C with 5 % CO_2_ in Roswell Park Memorial Institute medium (RPMI 1640, Carl Roth GmbH, Karlsruhe, Germany), supplemented with 10 % (*v*/v) FBS, 100 U/ml penicillin, 0.1 mg/ml streptomycin, and 0.1 % (v/v) non-essential amino acids (NEAA), which were obtained from PAN-Biotech GmbH (Aidenbach, Germany).

#### 2.6.2 Cytotoxicity assay

Resazurin-based assays were conducted to assess the cytotoxicity of non-encapsulated ML-1 and the various liposomal formulations. The colon-26 cells were seeded into 96-well plates at a density of 10^5^ cells/ml with a total well volume of 100 μl. After a 24-h incubation at 37 °C with 5 % CO_2_ atmosphere, the culture medium was aspirated and replaced with 90 μl of fresh RPMI 1640. To evaluate cytotoxic effects, 10 μl of either non-encapsulated ML-1 or the different liposomal formulations were added to the wells at final drug concentrations ranging from 0.1 ng/ml to 200 ng/ml. Another 24-h incubation at 37 °C and 5 % CO_2_ was done, followed by the addition of 10 μl 0.15 mg/ml resazurin solution in Dulbecco's phosphate saline (Carl Roth GmbH & Co. KG, Karlsruhe, Germany). After a 2-h incubation period, the absorbance was measured at 570 nm (reference wavelength of 600 nm) with the microplate reader Tecan Sunrise™ (Tecan Trading AG, Männedorf, Switzerland). Relative cell viability for each sample was computed as the ratio of the sample absorbance to the absorbance of the control.

### 2.7 Statistical analysis

For statistical analysis, all experiments were performed in triplicate. Data are expressed as the mean ± standard deviation (S.D.). Significant differences were evaluated using Student's *t*-test or one-way ANOVA, followed by Tukey's HSD test. Depending on data distribution, either Pearson's correlation (r_p_) coefficient or Spearman's rank correlation coefficient (r_s_) was applied to assess correlations. A *p*-value of <0.05 was considered statistically significant.

## 3 Results and discussion

### 3.1 Liposomal characterization

The extrusion process of the samples was monitored by measuring the *Z*-Average and PDI after each extrusion step. The progression during extrusion process for every type of liposome, along with the data, can be found in the Supplementary material (Fig. S1.; Table S1 – Table S4). The final characteristics of the sterile filtered samples are summarized in Table 2. All samples achieved sizes <150 nm with PDI values <0.2. The values for the Z-Average and PDI of the drug-free control liposomes were significantly higher compared to the other formulations containing polyethylenglycol (PEG), which positively contributes to the reduction of liposomal size (Nogueira et al., 2013; Sriwongsitanont and Ueno, 2004). PEG2k induces maximal compression of the lipid bilayer at a concentration of 7 ± 2 mol%, leading to its dehydration and consequently reducing the size and enhancing the thermodynamic stability of the liposomal system (Garbuzenko et al., 2005; Priev et al., 2002). Zeta potential measurements revealed values close to neutrality, ranging from approximately −0.7 to −2.3 mV in the absence of lectin. No statistically significant differences were observed between ML-1 loaded and unloaded liposomes, indicating that ML-1 incorporation does not notably affect surface charge.

**Table 2:** Characterization of the size (*Z*-Average), polydispersity index (PDI) and zeta potential of non-galactosylated (Control / PEG2k) and galactosylated (Galx, x = 2.5; 5; 10) liposomes with or without encapsulated mistletoe lectin-1 (ML-1).

Type of formulation	Z-Average (nm)	PDI	Zeta Potential (mV)
Control	140.8 ± 11.5	0.199 ± 0.036	−1.35 ± 0.34
PEG2k	102.7 ± 4.7	0.077 ± 0.008	−2.34 ± 0.17
Gal2.5	103.9 ± 0.4	0.108 ± 0.018	−1.22 ± 0.50
Gal5	109.7 ± 0.8	0.109 ± 0.034	−0.67 ± 0.27
Gal10	115.4 ± 1.3	0.147 ± 0.024	−1.87 ± 0.43
Control + ML-1	120.6 ± 4.2	0.104 ± 0.025	−1.25 ± 0.40
PEG2k + ML-1	109.9 ± 4.0	0.103 ± 0.033	−2.10 ± 0.32
Gal2.5 + ML-1	112.6 ± 1.8	0.079 ± 0.034	−1.11 ± 0.53
Gal5 + ML-1	115.4 ± 1.4	0.096 ± 0.008	−1.31 ± 0.74
Gal10 + ML-1	122.3 ± 0.8	0.100 ± 0.017	−1.54 ± 0.24

### 3.2 Liposomal storage stabilities and trend analysis

The Z-Average and PDI of the various liposomal formulations with and without encapsulated ML-1 were measured weekly to assess their storage stabilities over 4 weeks at 4 °C. Trend analysis was done using a modified Student's *t*-test to evaluate size and sample homogeneity over time. The associated diagrams are included in the Supplementary material (Fig. S2 – S4) along with the data (Table S5 - Table S8). There were no significant changes in the Z-Average and PDI over the measured storage period for all liposomal types. The initial hypothesis was that an increase in Z-average and PDI may be possible due to potential cross-linking effects between galactosylated liposomes and ML-1. Mousavifar et al. (2025) previously demonstrated a 14-fold increase in Z-average and a 3-fold increase in PDI after incubating the mannose-binding lectin Concanavalin A (ConA) with mannosylated liposomes. The different behavior of ConA and ML-1-loaded liposomes is likely due to the fundamental structural differences between the two lectins: ML-1 contains only two galactose-binding sites, both located on the B-chain subunit, while ConA features four mannose-binding sites distributed across four subunits at the corners of its pyramid-shaped tetrameric structure (Krauspenhaar et al., 1999; PDB: 1CVN). This spatial arrangement in ConA greatly enhances accessibility and multivalent binding, promoting cross-linking. The use of 44-unit PEG spacers has been shown to sterically hinder lectin-liposome interactions, particularly with ricin, a galactose-binding lectin sharing approximately 52 % sequence homology with ML-1 (Shimada et al., 1997; Sweeney et al., 1998). In our study, despite using a similar PEG2k (44-unit) spacer conjugated to galactose, ML-1 binding to galactosylated liposomes was evident from elevated ML-1 recovery (Fig. 3a), while no agglutination occurred (Fig. S2 - S4). This suggests that PEG2k may reduce cross-linking potential under the tested conditions, while still allowing affinity-driven interactions between ML-1 and galactose moieties. Moreover, multiple parameters including anchor structure, spacer length, glycolipid type and molar ratio as well as the lectin itself influence sugar accessibility and recognition behavior (Faivre et al., 2003; Gelhausen et al., 1998; Tamiaki et al., 2006; Mauceri et al., 2015). Liposome size and lectin concentration both critically influence agglutination, with smaller liposomes and higher lectin levels promoting stronger agglutination (Wang et al., 2011). Taken together, these findings highlight that lectin recognition and agglutination are highly system-dependent, reflecting complex interactions between lectin structure, liposomal formulation, and physicochemical parameters. The combination of high ML-1 recovery (Fig. 3a) and stable formulation conditions supports the suitability of the galactosylated liposomal formulation. However, further mechanistic studies are needed to clarify conditions that enable or prevent ML-1-mediated agglutination and to identify formulation-related limitations, particularly regarding colloidal stability in drug delivery contexts.

### 3.3 Lipid recovery rate and bilayer distribution of PEG2k-Gal

**Fig. 1:**
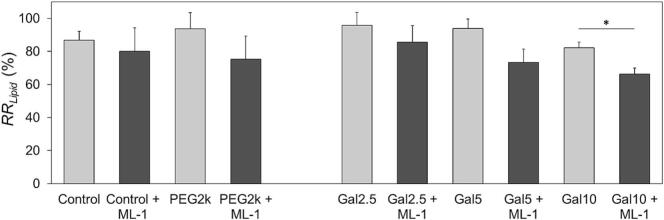
Total lipid recovery rate $\left({\mathrm{RR}}_{\mathrm{Lipid}}\right)$ for different liposomal formulations after manufacturing process and sterile filtration. Galactosylated liposomes (Galx, x = 2.5; 5; 10) were compared to non-galactosylated formulations (Control / PEG2k) with or without encapsulated mistletoe lectin (ML-1). The bars represent the mean ± S.D.; *n* = 3. Statistical analysis was done between each type of formulation with and without ML-1 using Student's *t*-test (*p < 0.05).

To evaluate the efficiency of the manufacturing process, the various liposomal formulations were characterized in terms of their total lipid recovery rate ${\mathrm{RR}}_{\mathrm{Lipid}}$ after final sterile filtration of the samples (Fig. 1). Data for the comparison of the ${\mathrm{RR}}_{\mathrm{Lipid}}$ are included in the Supplementary material (Table S9). The resulting ${\mathrm{RR}}_{\mathrm{Lipid}}$ values between each type of formulation with and without encapsulated lectin were not statistically different, except for the formulation of PEG2k-Gal10 + ML-1 compared with its drug-free variant PEG2k-Gal10.

**Fig. 2:**
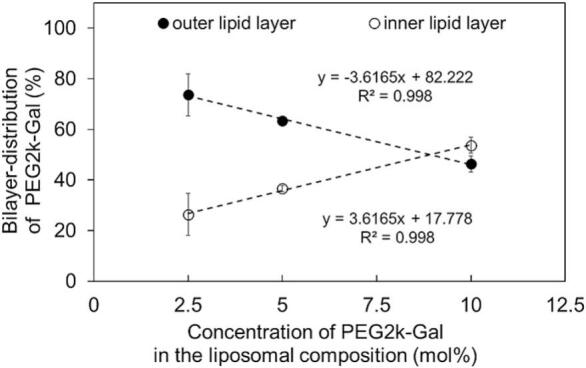
Percentual distribution of the galactosylated lipid PEG2k-Gal within the bilayer structure depending on its molar concentration in the liposomal formulation. The bars represent the mean ± S.D.; n = 3.

The liposomal samples containing the galactosylated lipid were subjected to enzymatic oxidation of the galactose residues, employing the colorimetric Amplex Red® Galactose/Galactose Oxidase Kit for the determination of its bilayer distribution. The molar concentration of the galactosylated lipid PEG2k-Gal in the liposomal composition correlated with the resulting bilayer distribution (Fig. 2). For the liposomes containing 2.5 mol% and 5 mol% of PEG2k-Gal, the lipid distribution $P_{\mathrm{out}}$ $/P_{\mathrm{in}}$ was calculated as 74 ± 8 % / 26 ± 8 % and 63 ± 1 % / 37 ± 1 %, respectively. The use of 10 mol% forms a shift to a more symmetrical arrangement of the PEG2k-Gal lipid with an $P_{\mathrm{out}}$ $/P_{\mathrm{in}}$ of 46 ± 3 % / 54 ± 3 %. Malewicz et al. demonstrated that the inclusion of 1 mol% galactosylceramide (GalCer) into pure DPPC (1,2-dipalmitoyl-*sn*-glycero-3-phosphocholin) or POPC (1-palmitoyl-2-oleoyl-glycero-3-phosphocholine) liposomes leads to a predominant localization of GalCer(∼ 70 %) in the inner lipid layer (Malewicz et al., 2005). Reducing the amount of POPC while adding 49.5 mol% sphingomyelin (SPM) to the liposomal formulation shifts the GalCer distribution to a more symmetrical arrangement, driven by intra- and intermolecular interactions, particularly hydrogen bonding (Malewicz et al., 2005; Mattjus et al., 2002). Another study focused on the transbilayer distribution of different galactolipids, including GalCer, monogalactosyldiacylglycerol (MGDG) and digalactosyldiacylglycerol (DGDG), integrated into unilamellar vesicles with a comparable size of 125 nm. The study also demonstrated that the distribution of galactose-containing structures in the inner and outer lipid layers is influenced by both the molar ratio and the specific galactose-containing structure. Additionally, the distribution was influenced by other membrane components, such as SPM, POPC, and DPPC, leading to an either symmetrical or asymmetrical arrangement of the investigated galactosylated structures (Fortelius and Mattjus, 2006). In the experiment conducted in this study, the galactosylated lipid PEG2k-Gal was used, which demonstrated a concentration-dependent effect on its transbilayer distribution. In addition to the influence of the PEG2k-Gal lipid's molar ratio itself, the presence of the other liposomal components may also modulate the proportion of the galactosylated lipid within the liposomal bilayer. Cholesterol, in particular, has the capacity to form hydrogen bonds with membrane components like phosphatidylcholines, which can significantly alter the bilayer's physical properties and organization. The extent of these changes depends on the concentration of cholesterol incorporated into the membrane structure (Bittman et al., 1994; Genova et al., 2018). Besides that, the inclusion of phytosterols, which are structurally similar to cholesterol, into model membranes induces a reorientation of the polar head groups of plant-derived galactolipids, such as MGDG and DGDG (Kergomard et al., 2022). In the investigated liposomes for the pharmaceutical preparation of ML-1, a high cholesterol content of 40 mol% was also incorporated, which likely promotes the formation of hydrogen bonds and may influence the alignment of galactose-containing molecules, as well as the overall membrane dynamics. To gain a more comprehensive understanding of the underlying mechanisms, future studies could investigate the effects of varying cholesterol fractions in additional formulations. This would offer a more detailed understanding of the orientation and transbilayer distribution of the galactose-containing lipid DSPE-PEG2k-Gal, as well as its influence on the structural properties and dynamic behavior of the bilayer.

### 3.4 Recovery rate of ML-1 after manufacturing process and purification of non-encapsulated protein

**Fig. 3:**
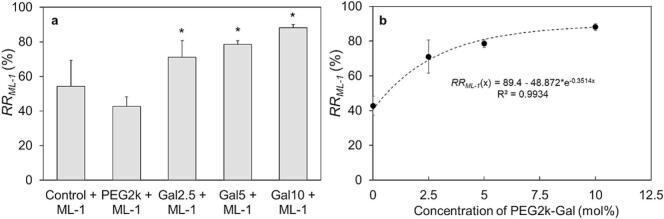
Recovery rate $\left({\mathrm{RR}}_{\mathrm{ML}-1}\right)$ of mistletoe lectin-1 (ML-1) after liposomal manufacturing process. The ${\mathrm{RR}}_{\mathrm{ML}-1}$ was compared between the non-galactosylated (Control + ML-1 / PEG2k + ML-1) and the galactosylated liposomes (Galx, x = 2.5; 5; 10 + ML-1) (a). Exponential saturation function of the ${\mathrm{RR}}_{\mathrm{ML}-1}$ and the molar concentration of the galactosylated lipid used in the liposomal composition (b). The bars and dots represent the mean ± S.D.; n = 3. Statistical analysis was done with one-way ANOVA, followed by Tukey's HSD test (*p < 0.05). Statistical correlation was assessed using Spearman's rank correlation coefficient, (r_s_ = 0.91; *p* = 0.0000471).

The galactosylated lipid PEG2k-Gal was integrated into the liposomal bilayer to investigate an adjusted composition for the efficient encapsulation of ML-1 triggered by its galactose-specific domain. First, the ${\mathrm{RR}}_{\mathrm{ML}-1}$ was determined by measuring the total concentration of liposomal ML-1 after purification from free non-encapsulated protein (Fig. 3a). In comparison to the initially used drug concentration ($c_{\mathrm{ML}-1})$, the ${\mathrm{RR}}_{\mathrm{ML}-1}$ for the galactose-free formulations reached values of 54.2 ± 15.2 % (Control + ML-1) and 42.8 ± 5.4 % (PEG2k + ML-1). The galactosylated formulation resulted in ${\mathrm{RR}}_{\mathrm{ML}-1}$ values of approximately up to 2-fold higher compared to the non-galactosylated liposomes. As the mol% increases, the ${\mathrm{RR}}_{\mathrm{ML}-1}$ values were 71.1 ± 9.6 %, 78.6 ± 2.1 % and 88.1 ± 2.0 %, indicating an enhanced interaction between ML-1 with increased PEG2k-Gal content and therefore minimizable protein loss during manufacturing process. The relationship between the molar concentration of the galactosylated lipid (0, 2.5, 5, and 10 mol%) and the recovery rate of ML-1 (42.8 % to 88.1 %) was analyzed using the Spearman rank correlation coefficient. The analysis revealed a strong and statistically significant correlation (r_s_ = 0.91, *p* = 4.7 × 10^−5^). This indicates that the integration and increase of the molar fraction of the galactosylated lipid component significantly enhance ML-1 recovery after the manufacturing process (Fig. 3b)- Regarding the exponential function of the relationship of ${\mathrm{RR}}_{\mathrm{ML}-1}$ a maximum of the value can be extrapolated as *x*_*max*_ = 89.4 %. With the inclusion of 10 mol% PEG2k-Gal, the recovery rate of ML-1 has approached a plateau, indicating that further increases in galactose content do not provide additional benefits. Generally, glycolipids play a crucial role in stabilizing liposomes (Yi et al., 2023) and, as demonstrated here, enhance the interaction between the drug and liposomal components, reducing drug loss during production and purification. Increasing the molar fraction of PEG2k-Gal beyond this point may negatively impact liposome size. Zhu et al. reported that raising lactosylated lipid content from 10 mol% to 20 mol% caused a significant increase in liposomal size (Zhu et al., 2005). Based on these findings and the observed plateau in ${\mathrm{RR}}_{\mathrm{ML}-1}$ recovery, no further benefits are expected from increasing the PEG2k-Gal molar ratio in the formulation.

### 3.5 Liposomal surface-adsorption of ML-1

**Fig. 4:**
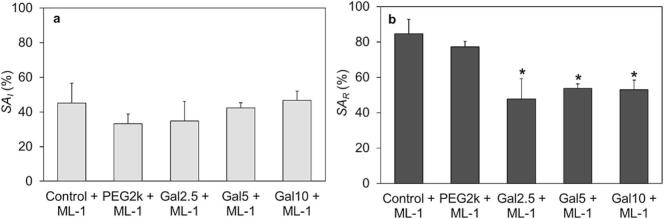
Percentual amount of surface-adsorbed $\left(\mathrm{SA}\right)\;$mistletoe lectin-1 (ML-1) for different liposomal formulations after manufacturing process. The concentration of the outer ML-1 is related to the initially used concentration (${\mathrm{SA}}_{I}$, a) and to the total recovered concentration of ML-1 after liposomal manufacturing (${\mathrm{SA}}_{R}$, b). The bars represent the mean ± S.D.; n = 3. Statistical analysis was done with one-way ANOVA comparing the non-galactosylated (Control + ML-1 / PEG2k + ML-1) with the galactosylated liposomes (Galx, x = 2.5; 5; 10 + ML-1), followed by Tukey's HSD test (*p < 0.05).

To quantify the protein adsorption of ML-1 on the liposomal surface after the purification process the surface adsorptions SA (in percent) for the liposomal compositions were calculated. The results are presented in Fig. 4, while complete SA values for all liposomal types are provided in the Supplementary material (Table S10). The comparison between the galactose-containing and the galactose-free liposomes showed no significant differences in the ${\mathrm{SA}}_{I}$ values of ML-1 (Fig. 4a). Pure DMPC liposomes interact with proteins primarily through hydrophobic forces, allowing the protein to penetrate into the lipophilic tail region of the lipid layer (Sabín et al., 2009). PEGylated lipids are asymmetrically distributed within the bilayer structure, with a strong preference for localization in the outer leaflet (Rovira-Bru et al., 2002). PEG is capable of engaging in specific interactions with proteins, with high-molecular-weight PEGs exhibiting stronger interactions due to their increased flexibility and ability to form multiple contact points (Wu et al., 2013; Wu et al., 2014). Aromatic amino acids such as tryptophan can mediate hydrophobic interactions with PEG, which are more pronounced with longer PEG chains as a result of their enhanced hydrophobic properties and contact area (Furness et al., 1998). In addition to direct protein interactions, PEG can influence the physicochemical properties of proteins, including their solubility, thermal stability, and structural integrity. Notably, alterations in secondary structure — such as changes in the α-helix content — have been observed depending on the molecular weight and concentration of PEG (Kumar et al., 2009). Cholesterol can also support the adsorption of several proteins on the liposomal surface e.g., IgGs and complement proteins (Caracciolo et al., 2013). Considering the variable possibilities of interactions between the liposomal components DMPC, cholesterol and PEG2k with proteins, as well as the asymmetrical bilayer distribution of PEG, the surface-adsorption behavior of liposomally formulated ML-1 can be explained. Regarding ${\mathrm{SA}}_{R}\;$in relation to the individual ${\mathrm{RR}}_{\mathrm{ML}-1}$ for each formulation, statistical differences were observed (Fig. 4b). In the case of the samples Control + ML-1 and PEG2k + ML-1, a major part of the recovered ML-1 is located on the surface of the liposomes, whereas the galactosylated Galx + ML-1 liposomes showed significantly lower levels of ${\mathrm{SA}}_{R}$. The results of the surface-adsorption of ML-1 related to ${\mathrm{RR}}_{\mathrm{ML}-1}$ demonstrate that the use of the galactosylated lipid alters the internal-to-external ratio of the lectin, shifting it inward compared to the Control + ML-1 and PEG2k + ML-1 samples.

In general, an ML-1 coating on the liposomal surface has the potential to induce endocytosis into tumor cells, enabled through the cell surface-targeting function of its B-chain (Beztsinna et al., 2018). Various lectin-modified nanoparticles have shown that the efficiency of cellular uptake is influenced by the surface density of the lectin and several lectin-drug conjugates showed enhanced cellular uptake in CRC mouse models, with the drug's efficacy correlating with the lectin's binding affinity (Kitaguchi et al., 2020; Russell-Jones et al., 1999; Yin et al., 2007). The principle of lectin-mediated cellular uptake has already demonstrated positive potential for the delivery of chemotherapeutic agents. Surface-modified liposomal formulations of DOX functionalized with lectins such as Lotus lectin (LTL), Peanut agglutinin (PNA), or rBC2LN lectin from *Burkholderia cenocepacia* have shown increased cellular uptake in various tumor cells, reduced systemic side effects, and enhanced cytotoxicity (Della Giovampaola et al., 2017; Kimura et al., 2021; Li et al., 2020). Consequently, the presence of ML-1 on the liposomal surface is expected advantageous for subsequent studies on cellular internalization, as it has the potential to act both as a facilitator of liposomal endocytosis and as an antitumoral agent for CRC.

### 3.6 Encapsulation efficiency of ML-1

**Fig. 5:**
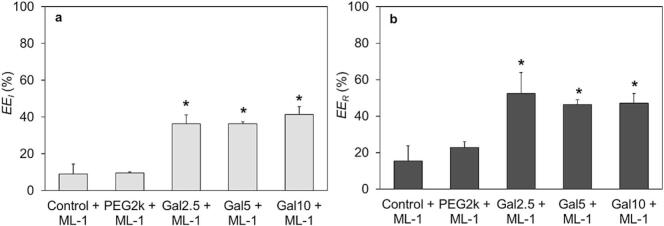
Encapsulation efficiency (EE) of mistletoe lectin-1 (ML-1) for different liposomal formulations after manufacturing process. The liposomal ML-1 is related to the initially used concentration (${\mathrm{EE}}_{I},$a) and to the total recovered concentration after liposomal manufacturing (${\mathrm{EE}}_{R},$b). The bars represent the mean ± S.D.; n = 3. Statistical analysis was done with one-way ANOVA comparing the non-galactosylated (Control + ML-1 / PEG2k + ML-1) with the galactosylated liposomes (Galx, x = 2.5; 5; 10 + ML-1), followed by Tukey's HSD test (*p < 0.05).

To evaluate the improved encapsulation by galactosylated lipids targeting ML-1, the encapsulation efficiency EE was determined. The ${\mathrm{EE}}_{I}$ for liposomally formulated ML-1 was calculated relative to the initial concentration used for the resuspension of the dried lipid film (Fig. 5a) and to the ${\mathrm{RR}}_{\mathrm{ML}-1}$ expressed as ${\mathrm{EE}}_{R}$ (Fig. 5b). The data for each parameter can be found in the Supplementary material (Table S10). In both instances, the galactosylated liposomes achieved significantly higher EE values, with results showing no significant variation among the different PEG2k-Gal concentrations (2.5 mol%, 5 mol% and 10 mol%) in the liposomal composition. The galactosylated formulations showed high values, with approximately 40 % for the ${\mathrm{EE}}_{I}$ and 50 % for ${\mathrm{EE}}_{R}$. These results demonstrate that utilizing individually tailored liposomal compositions, such as PEG2k-Gal as a functionalized liposomal component for the encapsulation of high molecular substances like ML-1, can significantly enhance the formulation's efficiency, by facilitating specific interactions. Earlier publications aiming to encapsulate ML-1 yielded comparable results for the lipid recovery rate ${\mathrm{RR}}_{\mathrm{Lipid}}$ as reached in this study, while the ${\mathrm{EE}}_{I}$ remained below 2 % (Manojlovic et al., 2008). Improvements were achieved through an optimized manufacturing process for the liposomes containing ML-1, resulted in an ${\mathrm{EE}}_{I}$ of 23 %, but without discrimination of encapsulated and surface-adsorbed ML-1 (de Matos et al., 2019). Therefore, this literature value must be compared to our ${\mathrm{RR}}_{\mathrm{ML}-1}$ results with around 70 % to 90 % (Fig. 4), demonstrating the significant improvement for the encapsulation of ML-1. A general comparison of encapsulation efficiencies for proteins reveals that Guimarães et al. encapsulated a similarly sized enzyme, L-Asparaginase (60 kDa), into PEGylated liposomes using the thin-film hydration method followed by extrusion steps, achieving a significantly lower ${\mathrm{EE}}_{I}$ of 10 % – 16 % after purification (de Souza Guimarães et al., 2022). For a smaller enzyme endolysin (15 KDa - 20 kDa) Gonçalves et al. achieved an ${\mathrm{EE}}_{I}$ of 24 % - 27 % using the extrusion method for production of enzyme-loaded liposomes which remains considerably lower than our affinity-mediated encapsulation efficiency of approximately 40 % for ML-1 - a lectin three times the size of endolysin (Gonçalves et al., 2024). Another commonly used method to achieve higher EEinvolves disrupting and resealing liposomes through repeated cycles of freezing and thawing. Freeze-thaw cycles have the ability to increase the EE but they also carry the risk of particle size growth, which can lead to increased sample inhomogeneity (Xu et al., 2011). Colletier et al. demonstrated that increasing the number of freeze-thaw cycles up to 20 enhances the EE of the enzyme acetylcholinesterase into liposomes, without any observable protein denaturation (Colletier et al., 2002). Another study also effectively confirmed the use of the freeze-thaw method for achieving efficient encapsulation of the antitumor lectin rCramoll into liposomal carriers. However, they observed a significant reduction in its hemagglutination activity after the third cycle, indicating limited applicability of the method due to possible negative impacts on the drug's functionality (da Cunha et al., 2016). Considering the mentioned disadvantages, the affinity-driven mechanism described here provides a distinctly better approach for efficient lectin encapsulation into liposomes compared to the freeze-thaw method, particularly by eliminating the thermal effects on the drug's activity. Studies on the general influence of factors affecting protein encapsulation were conducted by Hwang et al., where parameters such as molecular weight, buffer variation, lipid concentration, pH values, and the charge of phospholipids were identified as influencing factors for the EE (Hwang et al., 2012). Although an increase in the encapsulated concentration of the drug was observed, the EE did not reach values comparable to those achieved in this study. The use of affinity-functionalized lipids for encapsulation is therefore an extremely promising approach for achieving high EE through small modifications in the formulation of the pharmaceutical product.

### 3.7 Liposomal calculations

By calculating the increase factorIF for the EEfor the galactosylated samples compared to the two galactose-free liposomes (Control + ML-1 and PEG2k + ML-1) the impact of the demonstrated adjustment with the PEG2k-Gal lipid is highlighted (Table 3). Depending on the reference sample and its EE the utilization of 2.5 mol% - 10 mol% of PEG2k-Gal in the liposomal bilayer structure reached a 2-fold to 4-fold increase of the EE. This leads to a shift of the ratio $R_{{\mathrm{SA}}_{R}/{\mathrm{EE}}_{R}}$ from 9.1 ± 7.2 (Control + ML-1) and 3.5 ± 0.7 (PEG2k + ML-1) to almost equal distribution of the encapsulated and surface-adsorbed fraction of ML-1 with $R_{{\mathrm{SA}}_{R}/{\mathrm{EE}}_{R}}$ values of 1.0 ± 0.4, 1.2 ± 0.1 and 1.2 ± 0.2 with increased mol% of PEG2k-Gal.

**Table 3:** Increasing factors $\left(\mathrm{IF}\right)$ of encapsulation efficiency (EE) of mistletoe lectin-1 (ML-1) into liposomes containing 2.5 mol%, 5 mol% or 10 mol% of PEG2k-Gal (Galx, x = 2.5; 5; 10 + ML-1) compared to non-galactosylated samples Control + ML-1 and PEG2k + ML-1. IF was determined for the EE calculated in relation to the initially used concentration$\;\left({\mathrm{EE}}_{I}\right)$ and to the total recovered concentrations of ML-1$\;\left({\mathrm{EE}}_{R}\right)\;$after liposomal manufacturing process.

Type of formulation	IF of $\;{\mathrm{EE}}_{I}$	IF of $\;{\mathrm{EE}}_{R}$	IF of $\;{\mathrm{EE}}_{I}$	IF of $\;{\mathrm{EE}}_{R}$
	Compared to Control + ML-1		Compared to PEG2k + ML-1	
Gal2.5 + ML-1	4.0 ± 0.5	3.4 ± 0.7	2.3 ± 0.5	3.8 ± 0.6
Gal5 + ML-1	4.0 ± 0.1	3.0 ± 0.2	2.0 ± 0.1	3.8 ± 0.1
Gal10 + ML-1	4.5 ± 0.5	3.0 ± 0.3	2.1 ± 0.2	4.3 ± 0.6

In addition to assessing the encapsulated concentrations across the liposomal formulations, they were further described by calculating various characteristics, which are summarized in Table 4. The galactosylated liposomes contain a significantly higher number of ML-1 molecules, with a 3.5- to 5.5-fold increase of ML-1 molecules inside the liposomal core ($N_{\mathrm{In}})$, compared to $N_{\text{In}}$ for the galactose-free samples. The $N_{\mathrm{Total}}$ of recovered ML-1 increases with a higher mole fraction of PEG2k-Gal in the liposomal layer. The molecules on the outer surface ($N_{\mathrm{out}})$ are in a comparable range for all samples, with the PEGylated formulation having the lowest value. Pearson correlation analysis revealed a very strong positive correlation between the molar fraction of galactosylated lipid (0, 2.5, 5 and 10 mol%) and the number of ML-1 molecules encapsulated inside the liposomes $(N_{\mathrm{In}}$; r_p_ = 0.96, *p* = 0.042) as well as the total number of ML-1 per liposome ($N_{\mathrm{Total}}$; r_p_ = 0.98, *p* = 0.024). Both correlations are statistically significant. The external binding ($N_{\mathrm{Out}}$) show a moderate but non-significant correlation (r_p_ = 0.68, *p* = 0.32). In summary, increasing the molar galactosylation of the lipid membrane has a significant influence on the number of encapsulated molecules. This explains the observed increase in the total ${\mathrm{RR}}_{\mathrm{ML}-1}$ (Fig. 3b) as a function of mol% of the galactosylated lipid. In contrast, adsorption behavior does not appear to depend strongly on galactosylation, based on the calculated number of adsorbed ML-1 molecules. Fig. 4b shows a shift in the distribution of ${\mathrm{SA}}_{R}$, with a relative decrease in the adsorbed fraction for galactosylated formulations. During rehydration of the lipid film in the presence of dissolved ML-1, passive internal loading of the protein into liposomes occurs. Due to the relatively small internal aqueous liposomal volume ($V_{\mathrm{captured}}$: ∼ 1 %) compared to the external phase, the EE (Fig. 5) remains low. This is observed in both the Control + ML-1 and PEG2k + ML-1 samples, as reflected by their internal ($N_{\mathrm{In}}$) to external ($N_{\mathrm{Out}}$) ratio. As the majority of ML-1 molecules persists in the external medium after liposome formation, they can interact with the comparatively large outer volume (∼ 99 %) of the liposomes, leading to substantial surface association. This nonspecific binding is reduced upon incorporation of PEG2k ($N_{\mathrm{out}}=$57) indicating that PEGylation introduces steric hindrance that limits protein adsorption compared to the Control + ML-1 ($N_{\mathrm{out}}=$78). In parallel, incorporation of galactose-functionalized lipids enhances ML-1 accumulation inside the liposomes. During liposome formation, galactose units promote affinity-mediated lectin-sugar interactions, thereby increasing their local concentration at the bilayer interface and thus facilitating encapsulation. In addition, galactose residues on the outer surface contribute to a moderate increase in ML-1 surface binding. The number of surface-bound proteins can increase after the formation of the liposomal structure through additional ML-1 attachment via both non-specific and specific interactions, forming a dense protein corona.

**Table 4:** Calculations for the characterization of non-galactosylated (Control + ML-1 / PEG2k + ML-1) and galactosylated formulations (Galx, x = 2.5; 5; 10 + ML-1) with averages of: encapsulated mistletoe lectin-1 (ML-1), including the liposomal total inner volume per ml ($V_{\mathrm{captured}}$), number of lipid molecules per liposome $\left(N_{\mathrm{Lipid}}\right)$, number of liposomes per ml $\left(N_{\mathrm{Liposomes}}\right)$, number of encapsulated ML-1 molecules per ml ($N_{\mathrm{ML}-1})$ and the number of encapsulated ($N_{\mathrm{In}}$), surface-adsorbed ($N_{\mathrm{Out}}$), and total ML-1 molecules per liposome ($N_{\mathrm{Total}}$).

Type of formulation	$V_{\mathrm{captured}}$	$N_{\mathrm{Lipid}}$	$N_{\mathrm{Liposomes}}$	$N_{\mathrm{ML}-1}$	$N_{\mathrm{In}}$	$N_{\mathrm{Out}}$	$N_{\mathrm{Total}}$
Control + ML-1	9.9 μl	173,090	1.50 × 10^13^	2.19 × 10^14^	16	78	94
PEG2k + ML-1	9.0 μl	131,869	1.85 × 10^13^	2.32 × 10^14^	13	57	71
Gal2.5 + ML-1	10.9 μl	133,704	2.06 × 10^13^	8.77 × 10^14^	45	60	106
Gal5 + ML-1	12.0 μl	135,819	2.09 × 10^13^	8.76 × 10^14^	45	73	118
Gal10 + ML-1	10.6 μl	139,692	1.18 × 10^13^	9.97 × 10^14^	70	81	151

### 3.8 Antitumoral activity on colon carcinoma cells

**Fig. 6:**
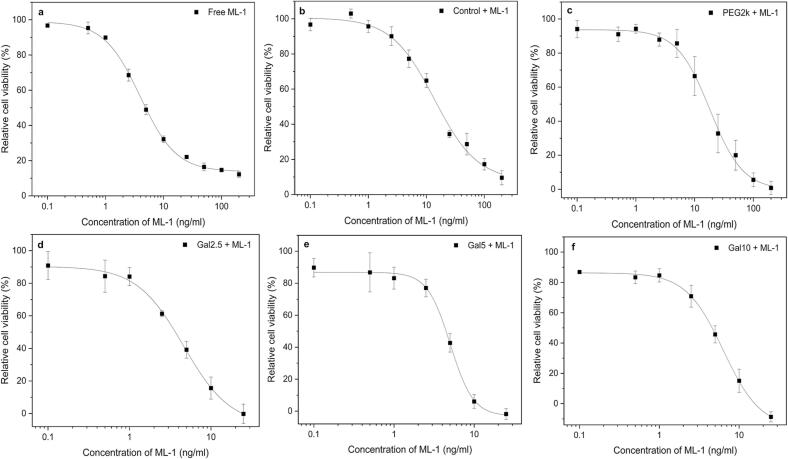
Dose-response curves of free and liposomal mistletoe lectin-1 (ML-1) on the colon carcinoma cell line colon-26. Free ML-1 (a) is compared to different liposomally formulated ML-1, where (b) shows liposomes without PEGylation (Control + ML-1) and (c) displays PEGylated liposomes (PEG2k + ML-1) with encapsulated ML-1. The results (d) - (f) show the galactosylated liposomes (Galx, x = 2.5; 5; 10 + ML-1) with encapsulated ML-1. The dots represent the mean + S.D.; n = 3.

Antitumoral activity was evaluated by the IC_50_ values for each liposomal type and for the free, non-encapsulated ML-1 on the murine colon carcinoma cell line colon-26 (Table 5) according to the corresponding dose-response curves shown in Fig. 6. The data for the different liposomal types are included in the Supplementary material (Table S11 and Table S12). For each liposomal treatment the IC_50_ was determined below 20 ng/ml, while the galactosylated liposomes are as effective as the free ML-1. Comparing the cytotoxicity profiles of all tested samples with the standard chemotherapeutic agent DOX for the treatment of CRC highlights the considerable therapeutic potential of ML-1. Hayashi et al. investigated free and liposomal formulated DOX on the colon-26 cell line, whereas the calculated IC_50_ values were 8.15 μg/ml and 16.23 μg/ml, respectively (Hayashi et al., 2013). These results indicate that achieving the same therapeutic in vitro effect with DOX required an approximately 2000- to 4000-fold higher dose compared to ML-1.

**Table 5:** IC_50_ values of free mistletoe lectin-1 (ML-1) and different liposomally formulated ML-1 using non-galactosylated (Control / PEG2k) and galactosylated (Galx, x = 2.5; 5; 10) formulations on the colon carcinoma cell line colon-26.

Type of formulation	IC_50_-value (ng/ml)
Free ML-1	4.0
Control + ML-1	13.8
PEG2k + ML-1	18.2
Gal2.5 + ML-1	4.6
Gal5 + ML-1	5.1
Gal10 + ML-1	6.1

The IC_50_ is 3.45-fold for the Control + ML-1 sample and 4.55-fold higher for the PEG2k + ML-1 liposomes compared to the free, non-encapsulated ML-1. The incorporation of PEG molecules plays an important role as it upregulates the half-life of liposomes after intravenous injection due to a decrease in uptake of the mononuclear phagocyte system (MPS) (Crosasso et al., 2000; Yang et al., 2007). The PEGylated membrane components preserve the efficacy of encapsulated substances by forming a barrier that reduces interactions with blood proteins. This decreases the in vivo opsonization, while improving circulation half-time of the drug delivery system (Gabizon et al., 2003, Pasut et al., 2015; Suk et al., 2016). On the other hand, PEGylation can hinder liposomal internalization via endocytotic pathway by limiting the interactions with the liposomal membrane and the cell surface, reducing intracellular drug level resulting in higher IC_50_ value (Li et al., 2015, Mishra et al., 2004).

The galactosylated liposomes with ML-1 achieve comparable IC_50_ results to those of the free drug with values below 7 ng/ml. The lower IC_50_ values observed for each galactosylated formulation suggest that both the liposomal uptake and the intracellular release need to be specifically targeted. Regarding the higher number of ML-1 molecules per galactosylated liposome ($N_{\mathrm{Total}}$) compared to the non-galactosylated formulations could explain the lower IC_50_ (Table 4). Each endocytic uptake of a single galactosylated liposome delivers more than twice the number of molecules compared to a non-galactosylated one. The extent to which the external surface-adsorbed lectin (SA) specifically facilitates or supports the cellular uptake and how this is influenced by its density requires further investigation in subsequent studies. In general, lectins are being investigated in the context of surface modifications of drug delivery systems to enable an active targeting strategy for enhanced cellular uptake (Silva, 2024). Lectin-modified drug carriers have shown improved interactions with glycosylated structures and enhanced cellular uptake. Their surface modifications were achieved through a covalent coupling of the lectin to the lipid membrane components (Bakowsky et al., 2008; Della Giovampaola et al., 2017; Ikemoto et al., 2016; Montisci et al., 2001). The method presented here offers a prospective distinct advantage regarding the possible targeting function of ML-1 next to its high antitumoral activity. The inner and outer part of ML-1 is supported via affinity-driven interactions regarding the galactosylated liposomes, where a dissociation from this state should be possible after internalization compared to a covalent linked ligand. Therefore, it is hypothesized that the outer ML-1 may initially function as an uptake inducer and, upon internalization and detachment from the liposomal surface, subsequently act as an antitumor agent. The results of this study clearly highlight the significant chance for new therapy options using the liposomally encapsulated ML-1. However, further investigations are required to elucidate and optimize the internalization process, including specifying the hypothesized dual functionality of the system in future studies.

## 4 Conclusion

Our study highlights the potential of galactosylated lipid components for the liposomal encapsulation of the highly antitumoral ML-1. Due to the specific binding affinity of its enzymatic domain to the galactose residues in the liposomal layers, the EEcan be enhanced by a factor of two to four. Simultaneously, the system exhibited high physicochemical stability without any observable agglutination effects. A positive correlation was observed between the number of calculated encapsulated molecules and the molar percentage of galactosylated lipid within the formulation, which additionally resulted in an enhanced recovery rate of ML-1. Cytotoxicity analysis of the optimized ML-1 formulations on colon-26 cells was performed, with galactosylated liposomes showing efficacy comparable to free ML-1 (IC_50_ < 7 ng/ml). In general, affinity-bound active ingredients (APIs) can provide a significant advancement in the pharmaceutical formulation of high molecular weight substances, especially with affinity of the API to bilayer ingredients. Specifically adjusted liposomes with high amounts of encapsulated ML-1 therefore represent a possible cancer therapy concept alone or in combination with cytostatic drugs. A dual effect could be achieved through the affinity-mediated incorporation of lectins into liposomes loaded with chemotherapeutic drugs, enhancing intracellular cytotoxicity through the synergistic action of two agents with different mechanisms. Beyond its applications in cancer therapy, the developed method can be adapted for the delivery of various relevant pharmaceutical enzymes e.g. in the field of inflammation, arthritis, cardiovascular diseases, or digestive disorders by functionalizing lipids with their specific API for an optimal delivery system for each.

## CRediT authorship contribution statement

**Josanna Kaufmann:** Writing – original draft, Visualization, Validation, Methodology, Investigation, Formal analysis, Data curation, Conceptualization. **Eray Cetin:** Validation, Investigation, Formal analysis, Data curation. **Tiana Kraus:** Investigation, Formal analysis, Data curation. **Harden Rieger:** Writing – review & editing, Resources. **Gero Leneweit:** Writing – review & editing, Supervision, Project administration, Funding acquisition.

## Declaration of competing interest

The authors declare that they have no competing financial interests or personal relationships that could have appeared to influence the work reported in this paper.

## Data Availability

Data supporting this article are included in the Supplementary material.

## Appendix A Supplementary data

Supplementary material Image 1
