# Supplementary material for: Galactosylated liposomes for targeted encapsulation and enhanced cytotoxicity of Mistletoe Lectin, an antitumoral type 2 ribosome-inactivating protein
Source: Int J Pharm X. 2025 Sep 12;10:100392. doi: 10.1016/j.ijpx.2025.100392 (PMC12475872; doi:10.1016/j.ijpx.2025.100392)
Supplement: Supplementary file 1 — Supplementary material [file mmc1.docx]

**Supplementary material**

**Galactosylated Liposomes for Targeted Encapsulation and Enhanced Cytotoxicity of Mistletoe Lectin, an Antitumoral
Type 2 Ribosome-Inactivating Protein**

Josanna Kaufmann ^a,b,c^, Eray Cetin ^a^, Tiana Kraus ^a^, Harden Rieger ^a,b^ , Gero Leneweit ^a, b, c, *^

^a^ ABNOBA GmbH, 75223 Niefern-Öschelbronn, Germany

^b^ Carl Gustav Carus-Institute, 75223 Niefern-Öschelbronn, Germany

^c^ Department of Chemical and Process Engineering, Institute of Mechanical Process Engineering and Mechanics, Karlsruhe Institute of Technology, 76131 Karlsruhe, Germany

* Correspondence: gero.leneweit2@kit.edu; Tel.: +49-7233-7043-101


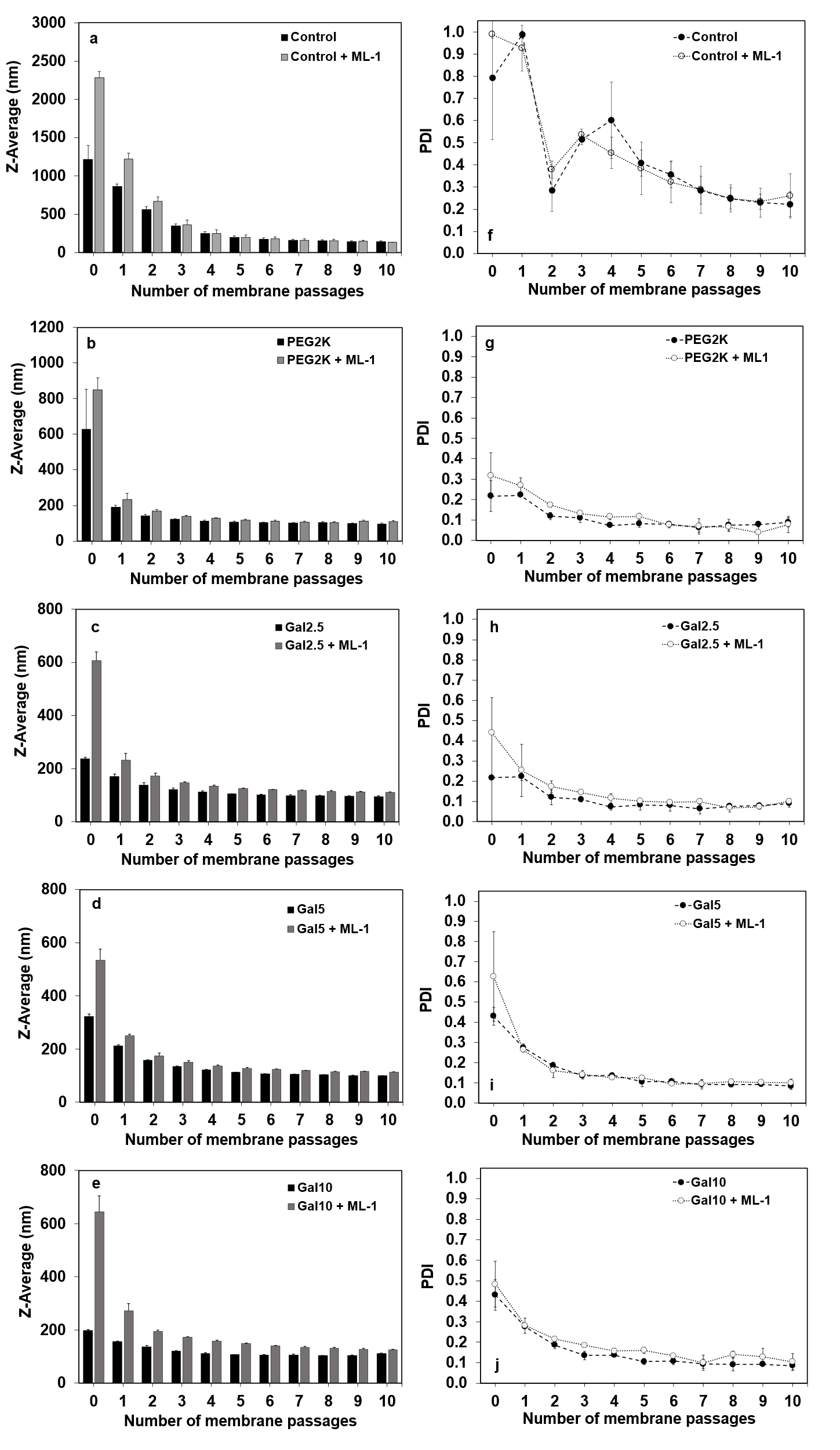


**Fig. S1.** Development of the liposomal size (Z-Average; a – e) and polydispersity index (PDI; f - j) during the extrusion process of non-galactosylated (Control / PEG2k) and galactosylated liposomes
(Galx, x = 2.5; 5; 10) with and without encapsulated mistletoe lectin-1 (ML-1) through a 100 nm track-etched membrane. The bars and dots represent the mean ± S.D.; n = 3.

**Table S1**

Liposomal size (Z-Average) of non-galactosylated (Control / PEG2k) and galactosylated
(Galx, x = 2.5; 5; 10) liposomes after each extrusion step through a 100 nm track-etched membrane;
n = 3.

|  | Z-Average Mean ± S.D. (nm) | | | | |
| --- | --- | --- | --- | --- | --- |
| Number of  membrane passages | **Control** | **PEG2k** | **Gal2.5** | **Gal5** | **Gal10** |
| 0 | 1215.1 ± 184.4 | 629.0 ± 222.7 | 238.2 ± 4.8 | 322.9 ± 8.9 | 197.4 ± 4.5 |
| 1 | 861.6 ± 34.5 | 189.6 ± 12.5 | 169.9 ± 10.3 | 212.8 ± 3.5 | 156.7 ± 2.1 |
| 2 | 562.5 ± 37.3 | 142.3 ± 9.3 | 138.6 ± 8.0 | 158.1 ± 2.4 | 136.4 ± 5.6 |
| 3 | 349.1 ± 25.2 | 122.1 ± 5.1 | 121.7 ± 4.7 | 134.8 ± 1.0 | 121.0 ± 0.9 |
| 4 | 249.1 ± 24.1 | 112.9 ± 4.4 | 113.7 ± 3.3 | 122.0 ± 1.2 | 111.9 ± 2.2 |
| 5 | 201.2 ± 16.2 | 107.9 ± 4.4 | 105.7 ± 0.4 | 112.9 ± 0.8 | 107.0 ± 1.0 |
| 6 | 176.2 ±14.5 | 104.7 ± 3.8 | 102.3 ± 1.9 | 107.5 ± 0.5 | 105.4 ± 1.6 |
| 7 | 161.4 ± 11.0 | 101.3 ± 3.7 | 98.8 ± 2.3 | 105.1 ± 1.4 | 105.9 ± 2.7 |
| 8 | 153.7 ± 11.1 | 103.5 ± 7.0 | 98.6 ± 2.2 | 103.2 ± 1.5 | 104.2 ± 0.4 |
| 9 | 144.2 ± 10.0 | 98.0 ± 4.9 | 95.7 ± 2.5 | 100.1 ± 1.4 | 104.0 ± 1.5 |
| 10 | 143.8 ± 13.7 | 97.0 ± 4.3 | 94.8 ± 2.9 | 99.9 ± 0.4 | 111.8 ± 0.5 |

**Table S2**

Liposomal size (Z-Average) of non-galactosylated (Control / PEG2k) and galactosylated
(Galx, x = 2.5; 5; 10) liposomes with encapsulated mistletoe lectin-1 (ML-1) after each extrusion step through a 100 nm track-etched membrane; n = 3.

|  | Z-Average Mean ± S.D. (nm) | | | | |
| --- | --- | --- | --- | --- | --- |
| Number of membrane passages | **Control**  **+ ML-1** | **PEG2k**  **+ ML-1** | **Gal2.5**  **+ ML-1** | **Gal5**  **+ ML-1** | **Gal10**  **+ ML-1** |
| 0 | 2283.6 ± 84.6 | 849.1 ± 177.1 | 606.0 ± 32.7 | 534.2 ± 42.3 | 644.7 ± 60.2 |
| 1 | 1220.6 ± 78.9 | 234.6 ± 27.7 | 232.2 ± 25.6 | 251.3 ± 5.1 | 271.4 ± 28.3 |
| 2 | 671.0 ± 52.8 | 168.1 ± 10.3 | 171.8 ± 11.7 | 173.7 ± 12.3 | 194.0 ± 6.0 |
| 3 | 359.5 ± 68.2 | 140.6 ± 5.4 | 146.7 ± 4.3 | 150.2 ± 5.6 | 172.4 ± 2.6 |
| 4 | 248.8 ± 49.5 | 128.1 ± 4.4 | 134.8 ± 3.8 | 136.2 ± 3.5 | 158.2 ± 3.2 |
| 5 | 201.2 ± 30.3 | 119.3 ± 2.9 | 125.5 ± 1.5 | 127.9 ± 4.0 | 148.4 ± 2.4 |
| 6 | 178.0 ± 26.5 | 112.3 ± 4.5 | 121.4 ± 0.5 | 124.2 ± 2.4 | 139.3 ± 2.0 |
| 7 | 164.3 ± 18.2 | 108-5 ± 3.0 | 118.2 ± 1.9 | 120.4 ± 0.6 | 135.3 ± 2.8 |
| 8 | 156.0 ± 16.9 | 105.5 ± 4.0 | 115.6 ± 2.6 | 115.6 ± 1.0 | 131.0 ± 3.3 |
| 9 | 147.6 ± 11.9 | 113.5 ± 10.3 | 112.4 ± 2.6 | 115.8 ± 0.1 | 128.1 ± 2.3 |
| 10 | 135.7 ± 2.4 | 111.1 ± 9.2 | 111.1 ± 1.9 | 113.0 ± 1.0 | 125.1 ± 2.5 |

**Table S3**

Polydispersity index (PDI) of non-galactosylated (Control / PEG2k) and galactosylated
(Galx, x = 2.5; 5; 10) liposomes after each extrusion step through a 100 nm track-etched membrane;
n = 3.

|  | PDI Mean ± S.D. | | | | |
| --- | --- | --- | --- | --- | --- |
| Number of membrane passages | **Control** | **PEG2k** | **Gal2.5** | **Gal5** | **Gal10** |
| 0 | 0.792 ± 0.278 | 0.217 ± 0.075 | 0.481 ± 0.004 | 0.431 ± 0.043 | 0.386 ± 0.020 |
| 1 | 0.989 ± 0.016 | 0.222 ± 0.007 | 0.206 ± 0.130 | 0.276 ± 0.018 | 0.282 ± 0.024 |
| 2 | 0.286 ± 0.094 | 0.120 ± 0.018 | 0.207 ± 0.028 | 0.186 ± 0.016 | 0.212 ± 0.011 |
| 3 | 0.515 ± 0.022 | 0.110 ± 0.021 | 0.181 ± 0.010 | 0.135 ± 0.011 | 0.174 ± 0.012 |
| 4 | 0.602 ± 0.172 | 0.073 ± 0.008 | 0.131 ± 0.022 | 0.137 ± 0.013 | 0.166 ± 0.006 |
| 5 | 0.408 ± 0.059 | 0.082 ± 0.016 | 0.157 ± 0.007 | 0.106 ± 0.023 | 0.169 ± 0.009 |
| 6 | 0.357 ± 0.061 | 0.079 ± 0.017 | 0.122 ± 0.010 | 0.107 ± 0.012 | 0.161 ± 0.011 |
| 7 | 0.285 ± 0.063 | 0.063 ± 0.023 | 0.104 ± 0.015 | 0.093 ± 0.024 | 0.195 ± 0.032 |
| 8 | 0.249 ± 0.047 | 0.075 ± 0.030 | 0.097 ± 0.022 | 0.091 ± 0.008 | 0.176 ± 0.005 |
| 9 | 0.229 ± 0.065 | 0.078 ± 0.013 | 0.111 ± 0.012 | 0.093 ± 0.010 | 0.195 ± 0.005 |
| 10 | 0.222 ± 0.057 | 0.089 ± 0.022 | 0.117 ± 0.013 | 0.085 ± 0.017 | 0.296 ± 0.033 |

**Table S4**

Polydispersity index (PDI) of non-galactosylated (Control / PEG2k) and galactosylated
(Galx, x = 2.5; 5; 10) liposomes with encapsulated mistletoe lectin-1 (ML-1) after each extrusion step through a 100 nm track-etched membrane; n = 3.

|  | PDI Mean ± S.D. | | | | |
| --- | --- | --- | --- | --- | --- |
| Number of membrane passages | **Control**  **+ ML-1** | **PEG2k**  **+ ML-1** | **Gal2.5**  **+ ML-1** | **Gal5**  **+ ML-1** | **Gal10**  **+ ML-1** |
| 0 | 0.988 ± 0.017 | 0.475 ± 0.283 | 0.439 ± 0.397 | 0.627 ± 0.220 | 0.484 ± 0.322 |
| 1 | 0.927 ± 0.103 | 0.233 ± 0.229 | 0.253 ± 0.018 | 0.264 ± 0.012 | 0.282 ± 0.012 |
| 2 | 0.380 ± 0.039 | 0.170 ± 0.135 | 0.174 ± 0.037 | 0.162 ± 0.036 | 0.216 ± 0.022 |
| 3 | 0.535 ± 0.026 | 0.143 ± 0.124 | 0.144 ± 0.013 | 0.142 ± 0.022 | 0.185 ± 0.040 |
| 4 | 0.454 ± 0.071 | 0.136 ± 0.093 | 0.115 ± 0.016 | 0.126 ± 0.009 | 0.157 ± 0.046 |
| 5 | 0.384 ± 0.119 | 0.096 ± 0.090 | 0.101 ± 0.024 | 0.125 ± 0.012 | 0.160 ± 0.020 |
| 6 | 0.322 ± 0.092 | 0.087 ± 0.088 | 0.095 ± 0.028 | 0.096 ± 0.001 | 0.133 ± 0.034 |
| 7 | 0.288 ± 0.105 | 0.074 ± 0.058 | 0.099 ± 0.025 | 0.097 ± 0.018 | 0.100 ± 0.031 |
| 8 | 0.248 ± 0.061 | 0.055 ± 0.077 | 0.068 ± 0.011 | 0.106 ± 0.014 | 0.141 ± 0.017 |
| 9 | 0.234 ± 0.035 | 0.026 ± 0.052 | 0.073 ± 0.015 | 0.102 ± 0.014 | 0.129 ± 0.012 |
| 10 | 0.260 ± 0.100 | 0.075 ± 0.082 | 0.100 ± 0.018 | 0.101 ± 0.018 | 0.104 ± 0.015 |


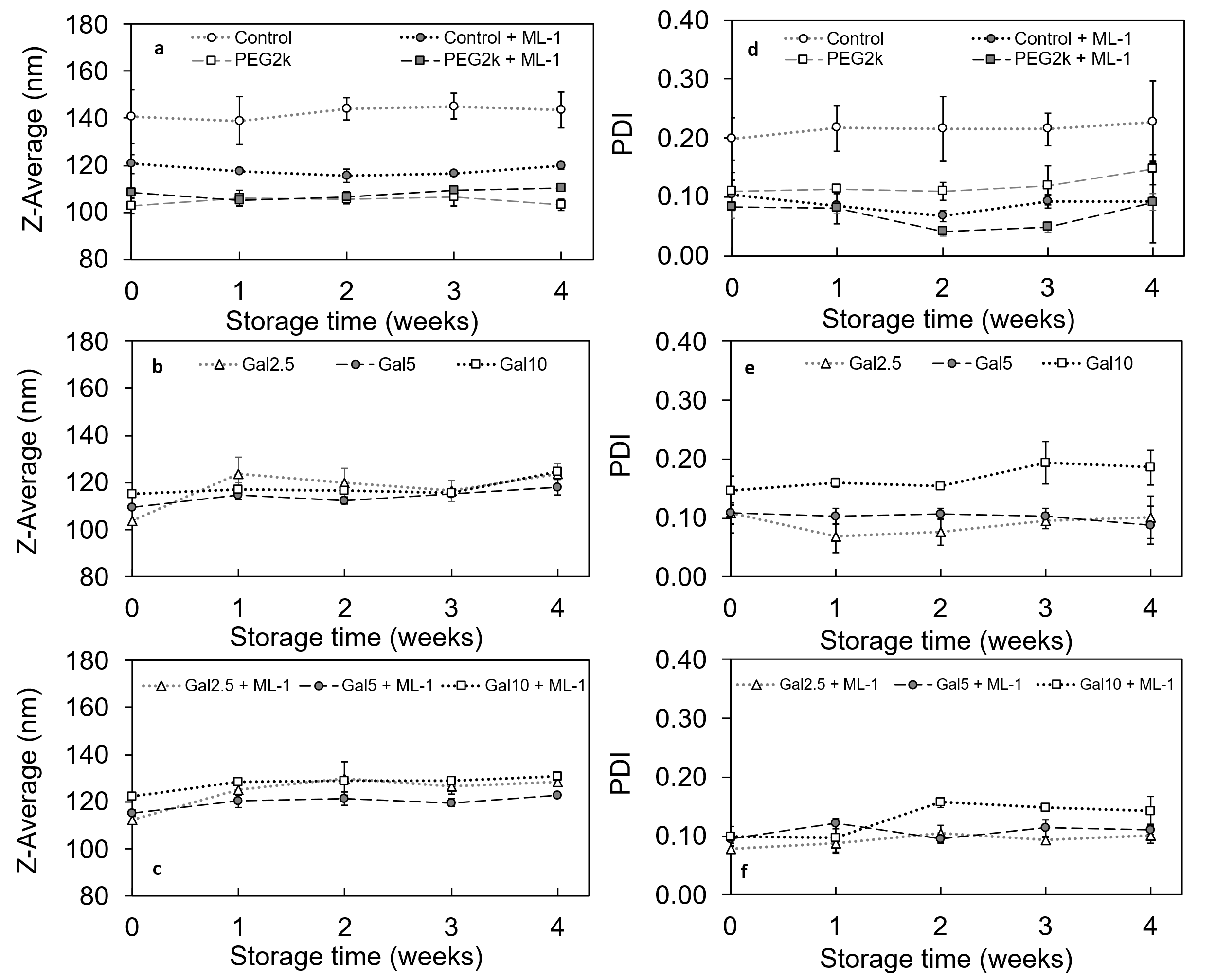


**Fig. S2.** Liposomal storage stabilities over 4 weeks at 4 °C. Development of the liposomal size
(Z-Average; a-c) and the polydispersity index (PDI; d-f) of non-galactosylated (Control / PEG2k) and galactosylated (Galx, x = 2.5; 5; 10) liposomes with or without encapsulated mistletoe lectin-1 (ML-1). The dots represent the mean ± S.D.; n = 3.

**
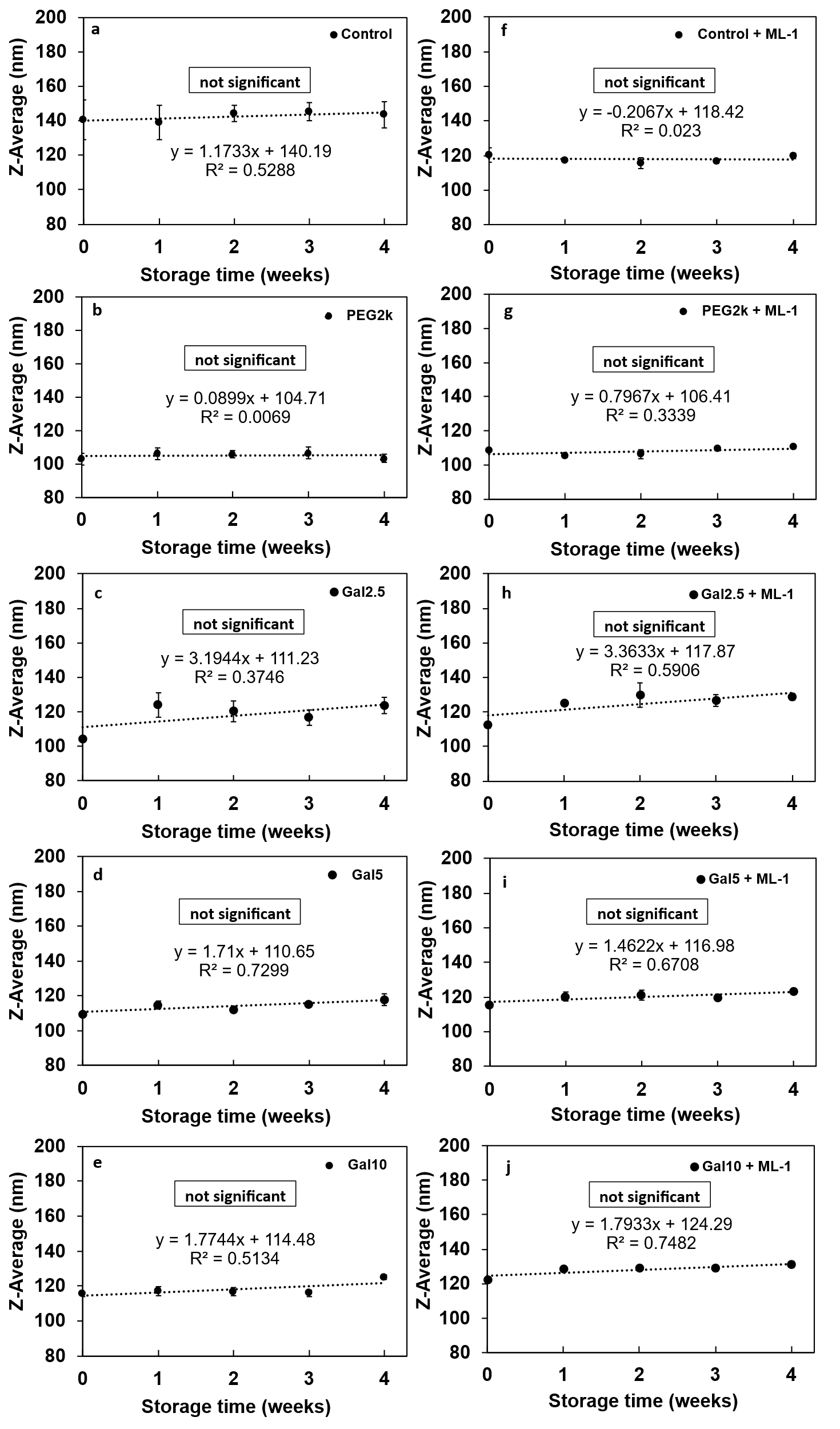
**

**Fig. S3.** Trend-analysis of the size (Z-Average) over 4 weeks at 4 °C for non-galactosylated (Control / PEG2k) and galactosylated (Gal, x = 2.5; 5; 10) liposomes with and without encapsulated mistletoe-lectin (ML-1) using modified Student´s t -test.

**
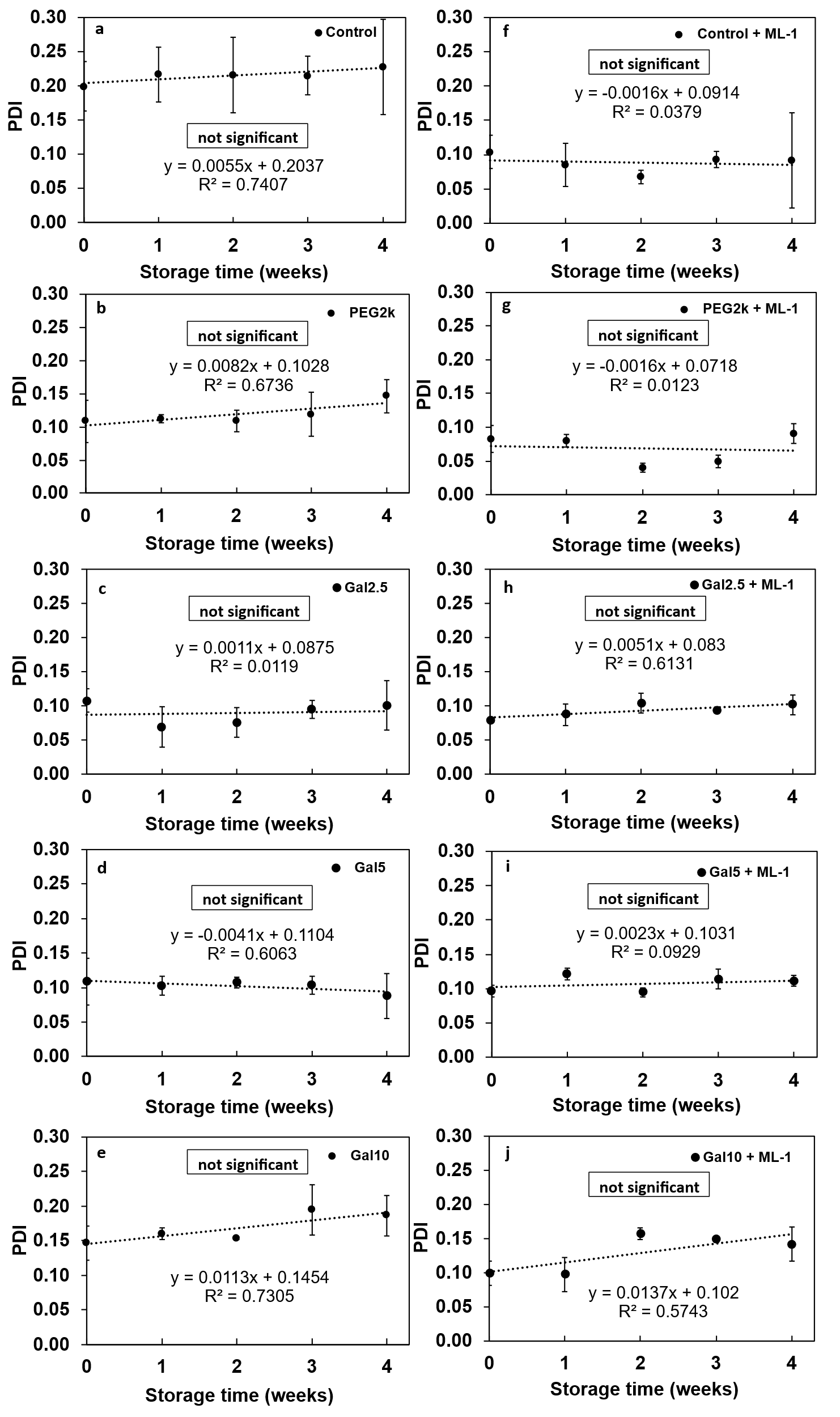
**

**Fig. S4.** Trend-analysis of the polydispersity index (PDI) over 4 weeks at 4 °C for non-galactosylated (Control / PEG2k) and galactosylated (Gal, x = 2.5; 5; 10) liposomes with and without encapsulated mistletoe-lectin (ML-1) using modified Student´s t -test.

**Table S5**

Progression of the liposomal size (Z-Average) over 4 weeks at 4 °C of non-galactosylated
(Control / PEG2k) and galactosylated (Galx, x = 2.5; 5; 10) liposomes; n = 3.

|  | Z-Average Mean ± S.D. (nm) | | | | |  |
| --- | --- | --- | --- | --- | --- | --- |
| Storage time (Weeks) | **Control** | **PEG2k** | **Gal2.5** | **Gal5** | **Gal10** | |
| 0 | 140.8 ± 11.5 | 102.9 ± 3.5 | 103.9 ± 0.4 | 109.7 ± 0.8 | 114.4 ± 1.3 | |
| 1 | 139.0 ± 10.0 | 106.2 ± 3.4 | 124.0 ± 7.0 | 114.9 ± 2.2 | 117.2 ± 2.6 | |
| 2 | 144.1 ± 4.7 | 105.8 ± 2.2 | 120.1 ± 6.0 | 112.3 ± 1.4 | 116.7 ± 2.5 | |
| 3 | 145.2 ± 5.4 | 106.4 ± 3.5 | 116.4 ± 4.5 | 115.5 ± 1.0 | 122.8 ± 2.5 | |
| 4 | 143.6 ± 7.6 | 103.2 ± 2.4 | 123.6 ± 4.6 | 118.0 ± 3.4 | 124.9 ± 1.2 | |

**Table S6**

Progression of the liposomal size (Z-Average) over 4 weeks at 4 °C of non-galactosylated
(Control / PEG2k) and galactosylated (Galx, x = 2.5; 5; 10) liposomes with encapsulated mistletoe
lectin-1 (ML-1); n = 3.

|  | Z-Average Mean ± S.D. (nm) | | | | |  |
| --- | --- | --- | --- | --- | --- | --- |
| Storage time (Weeks) | **Control**  **+ ML-1** | **PEG2k**  **+ ML-1** | **Gal2.5**  **+ ML-1** | **Gal5**  **+ ML-1** | **Gal10**  **+ ML-1** | |
| 0 | 120.6 ± 4.2 | 108.6 ± 0.3 | 112.6 ± 1.8 | 115.4 ± 1.4 | 122.3 ± 0.8 | |
| 1 | 117.4 ± 0.6 | 105.1 ± 1.1 | 125.2 ± 0.1 | 120.3 ± 2.8 | 128.3 ± 0.1 | |
| 2 | 115.6 ± 3.0 | 106.4 ± 2.7 | 129.7 ± 7.1 | 121.2 ± 2.8 | 128.9 ± 1.2 | |
| 3 | 116.6 ± 1.1 | 109.4 ± 0.8 | 126.7 ± 3.4 | 119.7 ± 1.6 | 128.8 ± 1.4 | |
| 4 | 119.9 ± 1.5 | 110.4 ± 1.1 | 128.7 ± 1.7 | 123.0 ± 1.0 | 131.1 ± 1.8 | |

**Table S7**

Progression of the polydispersity index (PDI) over 4 weeks at 4 °C of non-galactosylated
(Control / PEG2k) and galactosylated (Galx, x = 2.5; 5; 10) liposomes; n = 3.

|  | PDI Mean ± S.D. (nm) | | | | |  |
| --- | --- | --- | --- | --- | --- | --- |
| Storage time (Weeks) | **Control** | **PEG2k** | **Gal2.5** | **Gal5** | **Gal10** | |
| 0 | 0.199 ± 0.036 | 0.109 ± 0.032 | 0.108 ± 0.018 | 0.109 ± 0.034 | 0.147 ± 0.024 | |
| 1 | 0.217 ± 0.040 | 0.112 ± 0.006 | 0.069 ± 0.030 | 0.103 ± 0.014 | 0.160 ± 0.008 | |
| 2 | 0.216 ± 0.056 | 0.109 ± 0.016 | 0.076 ± 0.022 | 0.108 ± 0.008 | 0.153 ± 0.002 | |
| 3 | 0.215 ± 0.028 | 0.119 ± 0.033 | 0.095 ± 0.014 | 0.104 ± 0.013 | 0.194 ± 0.037 | |
| 4 | 0.227 ± 0.070 | 0.147 ± 0.025 | 0.101 ± 0.036 | 0.088 ± 0.033 | 0.186 ± 0.029 | |

**Table S8**

Progression of the polydispersity index (PDI) over 4 weeks at 4 °C of non-galactosylated
(Control / PEG2k) and galactosylated (Galx, x = 2.5; 5; 10) liposomes with encapsulated mistletoe
lectin-1 (ML-1); n = 3.

|  | PDI Mean ± S.D. (nm) | | | | |  |
| --- | --- | --- | --- | --- | --- | --- |
| Storage time (Weeks) | **Control**  **+ ML-1** | **PEG2k**  **+ ML-1** | **Gal2.5**  **+ ML-1** | **Gal5**  **+ ML-1** | **Gal10**  **+ ML-1** | |
| 0 | 0.104 ± 0.025 | 0.083 ± 0.020 | 0.079 ± 0.004 | 0.096 ± 0.009 | 0.100 ± 0.017 | |
| 1 | 0.085 ± 0.031 | 0.080 ± 0.009 | 0.088 ± 0.016 | 0.122 ± 0.008 | 0.098 ± 0.025 | |
| 2 | 0.068 ± 0.010 | 0.041 ± 0.007 | 0.104 ± 0.014 | 0.095 ± 0.007 | 0.158 ± 0.009 | |
| 3 | 0.093 ± 0.011 | 0.049 ± 0.009 | 0.093 ± 0.004 | 0.114 ± 0.015 | 0.149 ± 0.004 | |
| 4 | 0.092 ± 0.069 | 0.091 ± 0.014 | 0.102 ± 0.014 | 0.111 ± 0.008 | 0.142 ± 0.025 | |

**
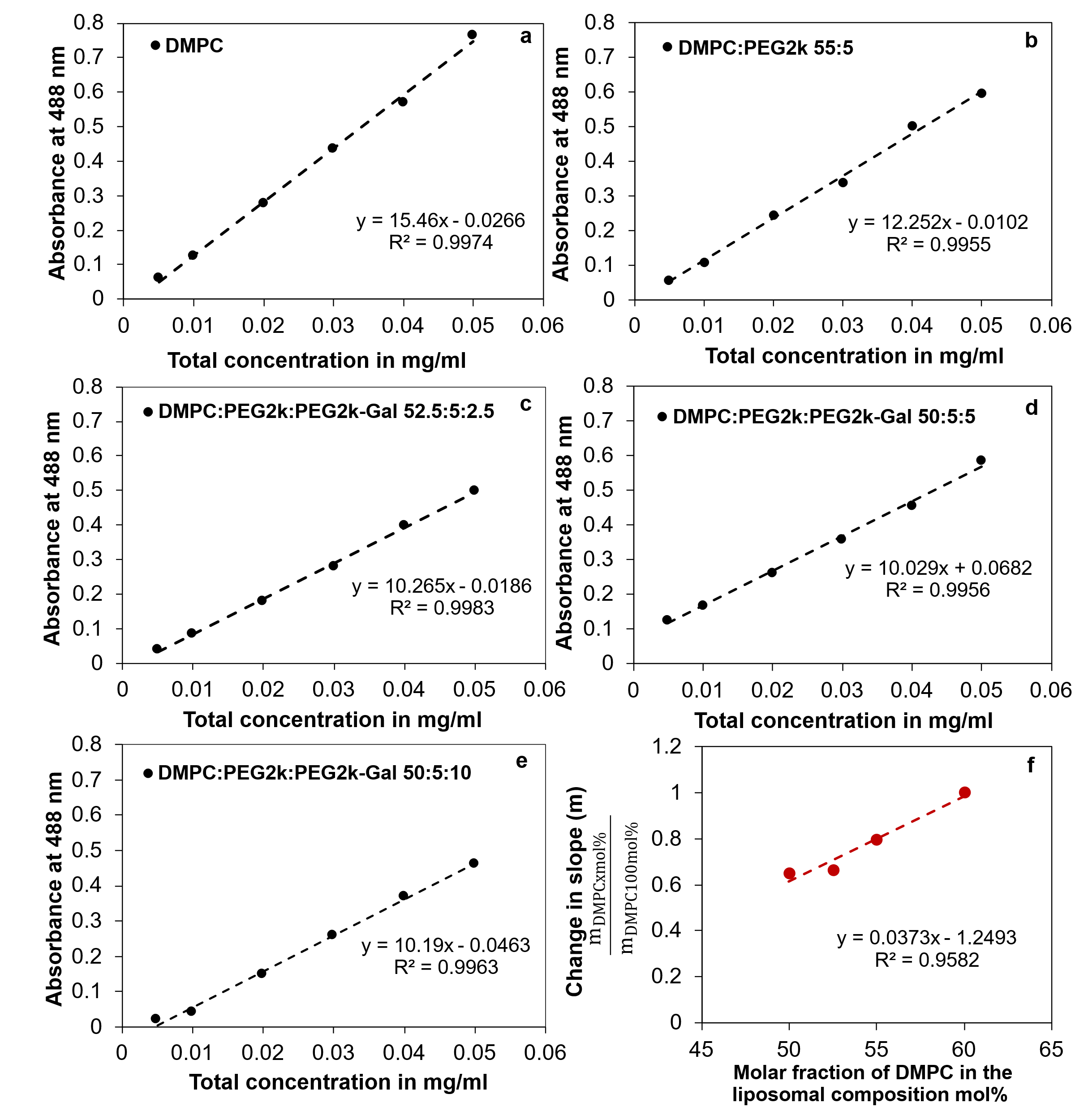
**

**Fig. S5.** Calibration curves for the Stewart assay using different compositions of phospholipids (a-e). Linear regression of the factor describing the change in the slope (m) of the calibration curve as a function of DMPC content (Pearson's r_p_ = 0.979, p = 0.02).

**Table S9**

Total lipid recovery rate $({RR}_{Lipid})$ of non-galactosylated (Control / PEG2k) and galactosylated (Galx, x = 2.5; 5; 10) liposomes with or without encapsulated mistletoe lectin-1
(ML-1) after the manufacturing process; n = 3.

| **Type of liposomal formulation** | $\boldsymbol{RR}_{\boldsymbol{Lipid}}$**(%)**  **Mean ± S.D.** |
| --- | --- |
| **Control** | 86.8 ± 5.3 |
| **PEG2k** | 93.6 ± 9.8 |
| **Gal2.5** | 95.8 ± 7.9 |
| **Gal5** | 93.9 ± 5.8 |
| **Gal10** | 82.3 ± 3.3 |
| **Control + ML-1** | 80.1 ± 14.2 |
| **PEG2k + ML-1** | 75.3 ± 13.9 |
| **Gal2.5 + ML-1** | 85.7 ± 9.9 |
| **Gal5 + ML-1** | 73.3 ± 8.1 |
| **Gal10 + ML-1** | 66.2 ± 3.8 |

**Table S10**

Encapsulation efficiencies ($EE$) and percentage of surface adsorption ($SA$) of mistletoe
lectin-1 (ML-1) encapsulated into non-galactosylated (Control / PEG2k) and galactosylated
(Galx, x = 2.5; 5; 10) liposomes. The percentual values are related to initial concentration of ML-1 at the beginning of the manufacturing process $({EE}_{I}$ and ${SA}_{I}$) and to the total recovered protein concentration after finishing the liposomal manufacturing $({EE}_{R}$ and ${SA}_{R}$); n = 3.

| **Type of liposomal**  **formulation** | ***EE_I_***  **(%)**  **Mean ± S.D.** | ***EE_R_***  **(%)**  **Mean ± S.D.** | ***SA_I_***  **(%)**  **Mean ± S.D.** | ***SA_R_***  **(%)**  **Mean ± S.D.** |
| --- | --- | --- | --- | --- |
| **Control + ML-1** | 9.1 ± 5.3 | 15.4 ± 8.3 | 45.1 ± 11.4 | 84.6 ± 8.3 |
| **PEG2k + ML-1** | 9.6 ± 0.6 | 22.8 ± 3.2 | 33.2 ± 5.6 | 77.2 ± 3.2 |
| **Gal2.5 + ML-1** | 36.4 ± 4.8 | 52.4 ± 11.6 | 34.7 ± 11.5 | 47.6 ± 11.5 |
| **Gal5 + ML-1** | 36.4 ± 1.0 | 46.3 ± 2.6 | 42.2 ± 3.2 | 53.7 ± 2.6 |
| **Gal10 + ML-1** | 41.4 ± 4.4 | 47.0 ± 5.4 | 46.7 ± 5.4 | 53.0 ± 5.4 |

**b**

**Table S11:** Dose-depending relative cell viabilities of colon-26 cells treated with free or
non-galactosylated liposomes (Control / PEG2k) with encapsulated mistletoe lectin-1 (ML-1); n = 3.

|  | Relative cell viability Mean ± S.D. (%) | | |
| --- | --- | --- | --- |
| Conc. ML-1  per well  (ng/ml) | **Free ML-1** | **Control**  **+ ML-1** | **PEG2k**  **+ ML-1** |
| 0.1 | 96.76 ± 0.62 | 96.73 ± 3.58 | 94.10 ± 5.12 |
| 0.5 | 95.37 ± 3.34 | 102.96 ± 2.58 | 91.04 ± 4.25 |
| 1 | 89.95 ± 1.12 | 95.69 ± 3.44 | 94.28 ± 2.57 |
| 2.5 | 68.60 ± 3.39 | 90.11 ± 5.31 | 87.91 ± 3.85 |
| 5 | 48.97 ± 2.82 | 77.26 ± 4.98 | 85.69 ± 8.21 |
| 10 | 32.16 ± 1.83 | 64.85 ± 4.01 | 66.49 ± 11.42 |
| 25 | 22.11 ± 0.56 | 34.43 ± 2.04 | 32.76 ± 11.33 |
| 50 | 16.41 ± 2.19 | 28.68 ± 6.14 | 19.99 ± 8.78 |
| 100 | 14.70 ± 0.20 | 17.27 ± 3.26 | 5.54 ± 4.04 |
| 200 | 12.19 ± 1.84 | 9.55 ± 4.00 | 0.81 ± 3.90 |

**Table S12:** Dose-depending relative cell viabilities of colon-26 cells treated with galactosylated liposomes (Galx, x = 2.5; 5; 10) with encapsulated mistletoe lectin-1 (ML-1); n = 3.

|  | Relative cell viability Mean ± S.D. (%) | | |
| --- | --- | --- | --- |
| Conc. ML-1  per well  (ng/ml) | **Gal2.5**  **+ ML-1** | **Gal5**  **+ ML-1** | **Gal10**  **+ ML-1** |
| 0.1 | 90.90 ± 8.58 | 89.80 ± 5.84 | 86.87 ± 0.60 |
| 0.5 | 84.34 ± 9.83 | 86.79 ± 12.24 | 83.34 ± 4.15 |
| 1 | 84.11 ± 5.54 | 83.19 ± 6.85 | 84.63 ± 4.39 |
| 2.5 | 61.27 ± 1.90 | 77.02 ± 5.50 | 70.88 ± 7.17 |
| 5 | 39.21 ± 5.18 | 42.71 ± 5.76 | 45.68 ± 5.64 |
| 10 | 15.65 ± 6.78 | 6.05 ± 4.31 | 15.12 ± 7.63 |
| 25 | -0.17 ± 5.97 | -1.82 ± 3.47 | -8.70 ± 3.33 |
